# The miR3367–lncRNA67–GhCYP724B module regulates male sterility by modulating brassinosteroid biosynthesis and interacting with Aorf27 in *Gossypium hirsutum*

**DOI:** 10.1111/jipb.13802

**Published:** 2024-11-11

**Authors:** Anhui Guo, Hushuai Nie, Huijing Li, Bin Li, Cheng Cheng, Kaiyun Jiang, Shengwei Zhu, Nan Zhao, Jinping Hua

**Affiliations:** ^1^ Laboratory of Cotton Genetics, Genomics and Breeding/Joint Laboratory for International Cooperation in Crop Molecular Breeding, Ministry of Education/Beijing Key Laboratory of Crop Genetic Improvement, College of Agronomy and Biotechnology China Agricultural University Beijing 100193 China; ^2^ Key Laboratory of Plant Molecular Physiology, Institute of Botany Chinese Academy of Sciences Beijing 100093 China

**Keywords:** brassinosteroids, *GhCYP724B*, *lncRNA67*, male sterility, mitochondrial Aorf27, upland cotton (*G. hirsutum*)

## Abstract

Cytoplasmic male sterile (CMS) lines play a crucial role in utilization of heterosis in crop plants. However, the mechanism underlying the manipulation of male sterility in cotton by long non‐coding RNA (lncRNA) and brassinosteroids (BRs) remains elusive. Here, using an integrative approach combining lncRNA transcriptomic profiles with virus‐induced gene silencing experiments, we identify a flower bud‐specific lncRNA in the maintainer line 2074B, lncRNA67, negatively modulating with male sterility in upland cotton (*Gossypium hirsutum*). lncRNA67 positively regulates cytochrome P274B (GhCYP724B), which acted as an eTM (endogenous target mimic) for miR3367. The suppression of *GhCYP724B* induced symptoms of BR deficiency and male semi‐sterility in upland cotton as well as in tobacco, which resulted from a reduction in the endogenous BR contents. GhCYP724B regulates BRs synthesis by interacting with GhDIM and GhCYP90B, two BRs biosynthesis proteins. Additionally, GhCYP724B suppressed a unique chimeric open reading frame (*Aorf27*) in 2074A mitochondrial genome. Ectopic expression of *Aorf27* in yeast inhibited cellular growth, and over expression of *Aorf27* in tobacco showed male sterility. Overall, the results proved that the miR3367–*lncRNA67–GhCYP724B* module positively regulates male sterility by modulating BRs biosynthesis. The findings uncovered the function of *lncRNA67–GhCYP724B* in male sterility, providing a new mechanism for understanding male sterility in upland cotton.

## Graphical abstract

In the fertile cotton (*Gossypium hirsutum*) line 2074B, the long non‐coding RNA lncRNA67 acted as an endogenous target mimic, regulating microRNA‐mediated cleavage of *GhCYP724B*, encoding a cytochrome P450 that promotes brassinosteroid biosynthesis. In the cytoplasmic male sterile line 2074A, GhCYP724B interacted with a unique chimeric open reading frame, resulting in male sterility.

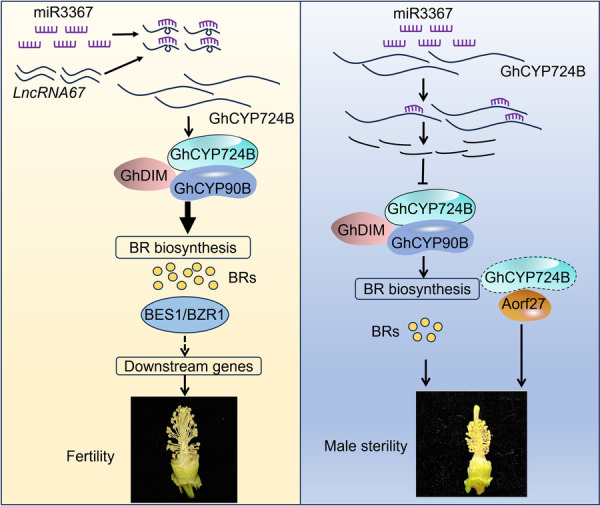

**Abbreviations**
AMS: aborted microspores
ARF: auxin response factors
BES1: brassinosteroid insensitive 1‐ems‐suppressor 1
BNP: binucleate pollen stage
BR: brassinosteroid
BRI1: brassinosteroid insensitive1
BRREs: BR response elements
BZR1: brassinazole resistant 1
CAT: catalase
CDS: coding sequences
CLCrV: cotton leaf crumple virus
CMS: cytoplasmic male sterility
CN: campestanol
CR: campesterol
CRISPR/Cas9: clustered regularly interspaced short palindromic repeats/CRISPR‐associated protein 9
CYP450s: cytochrome P450s
DEGs: differentially expressed genes
eTMs: endogenous target mimics
FPKM: fragments per kilobase of transcript per million mapped reads
GAs: gibberellin acids
GFP: green fluorescent protein
GO: gene ontology
lncRNAs: long non‐coding RNAs
JA: jasmonate
LC‐MS/MS: liquid chromatography and tandem mass spectrometry
MS1: male sterility 1
MT genes: mitochondrial functional genes
MYB: MYB domain protein
ORF: open reading frames
POD: peroxidase
RFP: red fluorescent protein
SOD: superoxide dismutase
SPL: squamosa promoter binding protein‐like
ssRNA‐seq: strand‐specific RNA sequencing
TDF1: tapetal development and function 1
TSI: tissue‐specific index
UNP: uninucleate pollen stage
VIGS: virus‐induced gene silencing
WT: wild type

## INTRODUCTION

The development of male reproductive organs in plants occurs via several major stages, including specification of the stamen primordia, development of the tapetum and microspore mother cells, meiosis, pollen coat deposition, and tapetum degeneration. Disrupting any of these steps may lead to male sterility (Huang et al., 2014; Zhang et al., 2021; Khan et al., 2023). Male sterility refers to the failure to produce dehiscent anthers, functional pollen, and viable male gametes, while the pistil can be pollinated by pollen from other plants (Chen and Liu, 2014; Chen et al., 2017). Male sterility is classified as cytoplasmic male sterility (CMS) or genic male sterility. When crossing plants for breeding, harnessing male sterility to avoid self‐pollination is preferred over time‐consuming manual emasculation, and CMS is widely utilized because it is stably inherited through the female parent (Gautam et al., 2023).

Cytoplasmic male sterility results from interactions between mitochondrial genes (MT genes) and coupled nuclear genes (Li et al., 2020; Nie et al., 2020), and may be mediated by non‐coding RNAs (ncRNAs) (Budak et al., 2020; Wang et al., 2022a; Nie et al., 2024). Long non‐coding RNAs (lncRNAs) are transcripts that are longer than 200 nucleotides (nt) and lack apparent coding capacity (Wang et al., 2017a; He et al., 2024); many such transcripts have known biological functions in plants (Palos et al., 2023). In rice (*Oryza sativa*), the lncRNA *TCONS_00057811* shows anther‐specific expression and is highly transcribed during meiosis. Plants overexpressing *TCONS_00057811* showed altered expression of the meiosis‐related gene *Meiotic Asynaptic Mutant 1* (*LOC_Os12g41350*), resulting in lower pollen fertility (29.70%) and seed setting (33%) compared to wild type (WT) plants (Li et al., 2020). Decreased expression of the pollen‐specific lncRNA *BcMF11* delayed tapetum degradation and inhibited proper pollen grain development in *Brassica campestris* (Song et al., 2013). LncRNAs influence the expression of CMS‐related genes in cotton (*Gossypium hirsutum*) by regulating the expression of members of the microRNA (miRNA) miR‐156, miR‐167, and miR‐159 families (Hamid et al., 2020). Most lncRNAs involved in inducing male sterility function by interacting with miRNAs and show tissue‐specific expression.

Brassinosteroids (BRs) are steroid phytohormones that function in plant reproductive development (Li and He, 2020; Nolan et al., 2020). BES1/BZR1, a crucial transcription factor that functions downstream of BR signaling, affects male fertility by regulating the expression of key reproductive genes (She et al., 2011; Yan et al., 2020). In BR‐related mutants, the dephosphorylation of BES1/BZR1 alters the expression of *NZZ*/*SPL*, *TDF1*, *AMS*, *MS1*, *MS2*, *MYB103*, and *AT3g23770*, leading to the abnormal development of the pollen cell outer walls, tapetum, and microspores, ultimately causing male sterility (Ye et al., 2010; Chen et al., 2019).

CYP724B, a member of the cytochrome P450 (CYP450) protein family, catalyzes the hydroxylation/oxidation reaction during BR biosynthesis (Carelli et al., 2019). A molecular evolutionary analysis of plant P450s showed that CYP724B and CYP90B are closely related and possess similar functions (Ohnishi, 2018). Both proteins are C‐22 hydroxylases that can convert campestanol (CN) to 6‐deoxocathasterone (6‐deoxoCT) and campesterol (CR) to (22S)‐22‐hydroxycampesterol (22OH‐CR). CYP724B affects plant fertility by regulating BR metabolism: *CYP724B1*‐overexpressing T_2_ transgenic rice exhibited a typical phenotype caused by high BR levels, whereas more than half of pollen grains were aborted due to a BR deficiency in *CYP724B1*‐deficient (RNA interference (RNAi)) rice (Zhu et al., 2015). The Arabidopsis (*Arabidopsis thaliana*) mutant *cyp90b1* exhibited symptoms of BR deficiency, including dwarfing and male sterility (Zhang et al., 2012), and overexpressing *CYP724A1*, a homolog of *CYP724B*, restored the *cyp90b1* mutant phenotype (Zhang et al., 2012). *Notched Belly Grain 4* (*NBG4*), which encodes CYP724B1, is specifically expressed in immature inflorescences in rice (Tong et al., 2018). The *nbg* mutant *A31*, obtained by deleting 10 bp of the seventh exon of *NBG4*, is a BR‐deficient mutant displaying dwarfing and fascicled spikelets in a “Y” or “W” shape. Exogenous BR treatment rescued lower germination ability of *A31* to a level comparable to the WT (Tong et al., 2018). Despite these insights, few studies have explored the effects of lncRNAs on BR metabolism in cotton.

Cotton is a major crop that benefits from heterosis, a process by which hybrid progeny of genetically dissimilar parents are more biologically fit than either parent. Cytoplasmic male sterility is a key tool for generating productive hybrid cotton lines for agriculture. The release of genome sequencing data from tetraploid cotton (Chang et al., 2024; Li et al., 2015; Zhang et al., 2015; Fang et al., 2017; Wang et al., 2017b; Hu et al., 2019; Ma et al., 2021) has provided new opportunities for functional genomics, which could facilitate the identification of high‐confidence lncRNAs.

To date, few lncRNAs related to male sterility in cotton have been investigated (Li et al., 2019, 2022; Zhang et al., 2019; Hamid et al., 2020). To identify functional lncRNAs that regulate male sterility in cotton, we performed whole‐transcriptome strand‐specific RNA sequencing (ssRNA‐seq) using flower buds of the CMS line 2074A and its maintainer line 2074B (Li et al., 2013). We identified *lncRNA67*, which is specifically expressed in flower buds, and confirmed its effect on male sterility using clustered regularly interspaced short palindromic repeats/CRISPR‐associated protein 9 (Cas9)‐mediated gene editing. lncRNA67 acts as an endogenous target mimic (eTM) of miR3367 to regulate *GhCYP724B*, which is highly expressed in the flower buds of fertile lines and encodes a 22α hydroxylase that controls a rate‐limiting step in the BR biosynthetic pathway. Knocking out *lncRNA67* or *GhCYP724B* blocked BR biosynthesis and led to male sterility, as GhCYP724B interacts with GhCYP90B and GhDIM to promote pollen fertility. Moreover, *Aorf27*, a unique chimeric open reading frame (ORF) in the mitochondrial genome of the sterile line 2074A (Li et al., 2018a), was toxic to yeast and caused male sterility in *Nicotiana tabacum*. These results indicate that the lncRNA67–GhCYP724B module regulates male sterility by suppressing *Aorf27* expression and affecting BR biosynthesis, thereby disrupting pollen development.

## RESULTS

### Identification of lncRNAs associated with male sterility in upland cotton

Cytological analysis of pollen development revealed no significant differences in flower buds between the CMS line 2074A and its maintainer line 2074B at the sporogonium stage (Figure S1A, F). During the period from pollen mother cell to late uninucleate pollen (UNP) formation, the tapetum cells degraded prematurely and microspores gathered in 2074A. Subsequently, the expanded tapetum cells invaded the inner chamber and microspores were squeezed into the central area of the anther (Figure S1B, C). The release of microspores from tetrads and the production of uninucleate microspores were inhibited, resulting in the rupture and death of microspores at the late uninuclear stage. Consequently, at the binucleate pollen stage, no intact microspores were observed in 2074A, and these plants failed to produce pollen grains (Figure S1D, E). By contrast, the microspore development of 2074B was normal, producing cells with a regular round shape that gradually accumulated storage materials to produce fertile pollen grains (Figure S1F–J).

We measured the nutrient contents and antioxidant enzyme activities in flower buds from pollen mother cell to late UNP formation. The total soluble sugar and proline contents were significantly lower in 2074A than in 2074B. By contrast, no significant difference in soluble protein content was detected. The level of malondialdehyde, the main product of lipid peroxidation, was significantly higher in 2074A; however, the activities of antioxidant enzymes, including superoxide dismutase (SOD), peroxidase (POD), and catalase (CAT), were significantly higher in 2074B (Figure S2). These findings indicate that the stage from pollen mother cell to late UNP formation is the major period of pollen abortion in 2074A.

**Figure 1:**
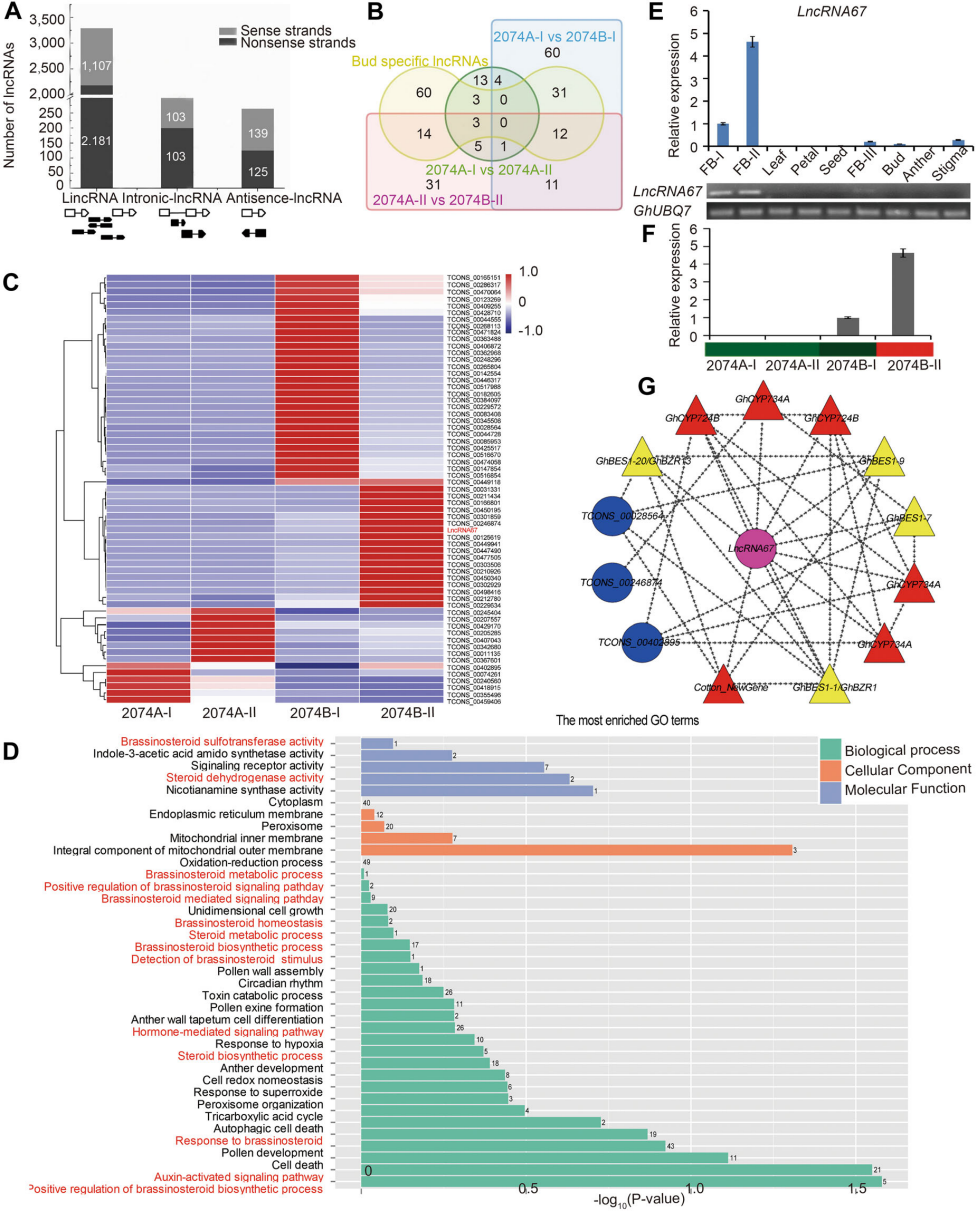
Identification of long non‐coding RNAs (lncRNAs) related to cytoplasmic male sterility(CMS) in upland cotton **(A)** Classification of 3,855 lncRNAs in upland cotton. **(B)** Venn diagram of tissue‐specific lncRNAs and different expression lncRNAs. Red words: 63 lncRNAs specifically and differentially expressed in the flower bud in sterile line 2074A and maintainer line 2074B. **(C)** Expression pattern of 63 lncRNAs specifically and differentially expressed in the flower bud in 2074A and 2074B. 2074A‐I: flower buds at sporogonium stage of CMS line 2074A (<1.5 mm); 2074A‐II: flower buds at pollen mother cells to late uninucleate pollen (UNP) stage of CMS line 2074A (1.5–8.0 mm); 2074B‐I: flower buds at sporogonium stage of CMS maintainer line 2074B (<1.5 mm); 2074B‐II: flower buds at pollen mother cells to late UNP stage of CMS maintainer line 2074B (1.5–8.0 mm). **(D)** Functional analysis of the lncRNA target genes; red letters represent the Gene Ontology terms related to brassinosteroids (BRs) metabolism. **(E)** The expression pattern of lncRNA67 in different tissues in upland cotton. **(F)** The expression pattern of lncRNA67 in 2074A and 2074B. **(G)** An lncRNA67‐centered view of the interplay between BR‐related genes. The magenta circle represents lncRNAs significantly interconnected with BR genes; the blue circle represents bud‐specific lncRNAs. The red and yellow triangle represent the BR biosynthesis genes and BR signaling genes, respectively.

To systematically identify lncRNAs related to male sterility in upland cotton, we performed whole‐transcriptome ssRNA‐seq of flower buds from 2074A and 2074B. We identified 3,855 high‐confidence lncRNAs in the four samples (Figure 1A; Table S1), 85.3% of which were located in intergenic regions (lincRNAs), with most derived from the sense strand (Figure 1A). The lncRNA transcripts were shorter and had fewer exons than the messenger RNAs (mRNAs) (Figure S3A, B). More exons in mRNAs resulted in shorter exon lengths (~235 nt) compared to lincRNAs (~436 nt), antisense‐lncRNAs (~480 nt), and intronic‐lncRNAs (~418 nt) (Figure S3C). The abundance of lncRNAs was lower than that of mRNAs in the RNA‐seq samples (Figure S3D). We constructed a Circos plot, which shows that all three types of cotton lncRNAs and mRNAs were evenly distributed across the chromosomes, whereas most miRNAs (Nie et al., 2018) were transcribed from loci much closer to telomeres (Figure S3E).

To investigate the functions of lncRNAs in upland cotton, we used the tissue specificity index (TSI) to evaluate tissue‐specific expression. We examined the expression patterns of the 3,855 lncRNAs in 12 upland cotton tissues (Table S1). Overall, 307 lncRNAs (8.0%) showed tissue‐specific expression, and a large proportion of lncRNAs (3,548; ~92.0%) were expressed in all tissues (Figure S4A, B; Table S2). Among the 307 tissue‐specific lncRNAs, approximately 40% (123 of 307) were highly enriched in flower buds (FB‐I and FB‐II). A total of 187 lncRNAs were differentially expressed between 2074A and 2074B (Figure S4C). The results of quantitative reverse transcription polymerase chain reaction (qRT‐PCR) were largely consistent with the expression levels identified by RNA‐seq (*R* ^2^ = 0.7708, Figure S5A), validating the accuracy of the RNA‐seq results. Of the 187 lncRNAs that were differentially expressed between 2074A and 2074B, 63 were specifically expressed in flower buds (Figure 1B) at either the sporogonium stage or the stage from pollen mother cell to late UNP formation (Figure 1C; Table S3). qRT‐PCR and semi‐quantitative PCR indicated that the whole‐transcriptome sequencing and bioinformatics analyses were reliable and highlighted some genes that might be involved in CMS (Figure S5A–C).

Based on cis‐ and trans‐regulatory patterns, 503 protein‐coding genes were identified as targets of the 63 lncRNAs (Figure S6). Gene Ontology (GO) analysis showed that these genes were involved in several BR‐related pathways, including BR biosynthetic processes, BR metabolic processes, and BR homeostasis (Figure 1D). These results suggest that lncRNAs might play important roles in pollen development by regulating BR metabolism.

To further elucidate the interplay between protein‐coding and non‐coding RNAs in the BR pathway, we examined the co‐expression of the 63 lncRNAs and the BR‐related genes. To determine whether these genes were associated with the lncRNAs, we integrated the lncRNA and mRNA expression data and analyzed the correlations. Any lncRNA–mRNA combinations with correlation coefficients >0.95 or <−0.95 were retained. We ultimately identified 54 BR‐related genes as potential targets of 54 lncRNAs (Figure S6; Table S4). Among the 54 lncRNAs, the expression levels of 10 were significantly correlated with the expression of BR‐related genes (Table S4; Figure S6; pink circles), including *lncRNA67*, *TCONS_00207557*, and *TCONS_00165151* (Figure S5C). Among the 10 lncRNAs, *lncRNA67* was highly expressed in flower buds of the maintainer line 2074B but was not expressed in CMS line 2074A, suggesting it might play an important role in male sterility (Figure 1E, F). Moreover, the expression of *lncRNA67* was significantly correlated with that of six BR‐biosynthesis genes and three BR signal transduction genes, including *GhCYP724B*, *GhCYP734A*, and *GhBES1* (Figure 1G).

### Long non‐coding RNA67, an eTM of miR3367, positively regulates *GhCYP724B* expression

Combined with the previously identified correlations between miRNAs and CMS (Nie et al., 2018), we identified 38 lncRNAs that could be pre‐miRNAs and generate 47 types of miRNAs, as well as 359 lncRNAs that were targeted by 187 miRNAs and 23 lncRNAs that act as potential eTMs for 18 miRNAs (Tables S5–S7).

**Figure 2:**
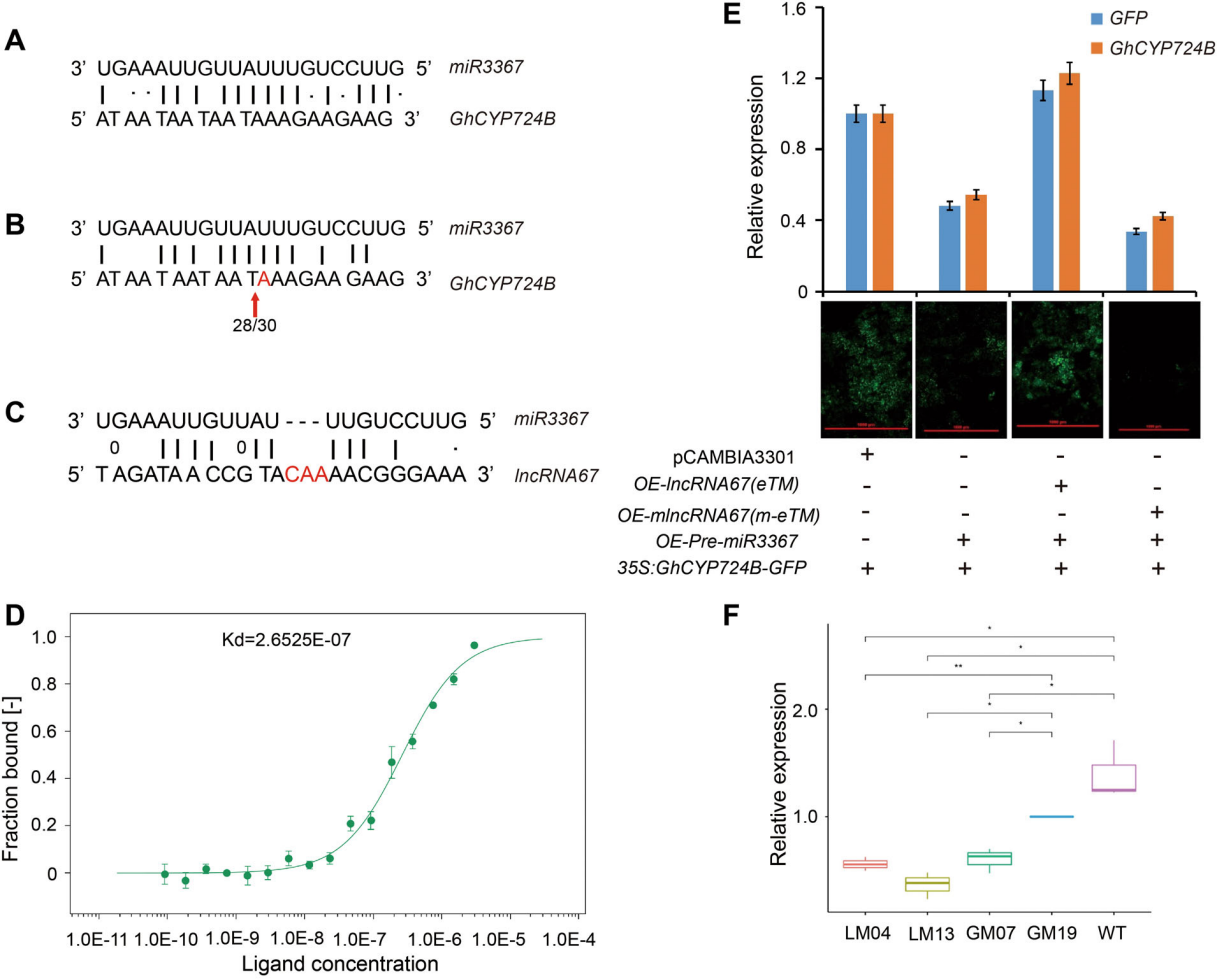
***lncRNA67*** **regulates the expression of** ***GhCYP724B*** **by acting as an eTM (endogenous target mimic) of *microR3367* (*miR3367*) in upland cotton** **(A)** Predicted base‐pairing interactions between *miR3367* and *GhCYP724B*. **(B)** Cleavage sites in *GhCYP724B* transcripts confirmed by rapid amplification of complementary DNA (cDNA) 5′ ends (5′‐RACE) analysis. Arrows indicate the cleavage sites, and numbers below the arrows show frequency of clones with matching of this site out of the total sequenced clones. **(C)** Base‐pairing prediction of *lncRNA67* and *miR3367*; the bases underlined in red represent the mutated base sites in *mlncRNA67*. **(D)** Binding affinity of *miR3367* and *long* *non*‐*coding* *RNA67* (*lncRNA67*) were determined using microscale thermophoresis (MST) analysis. The *K*_*d*_ (dissociation constant) of lncRNA67 is 2.6525E‐07M. **(E)** Tobacco transient transformation to verify the interaction between *miR3367*, *lncRNA67* and *GhCYP724B*. The expression abundances of *GhCYP724B* and *GFP* were detected using reverse transcription – quantitative polymerase chain reaction (RT‐qPCR). The GFP (green fluorescent protein) intensity was evaluated using a fluorescence microscope. **(F)** The expression pattern of *miR3367* in the flower bud of *lncRNA67* mutants (LM04, LM13), *GhCYP724B* mutants (GM07, GM19) and wild type cotton. *, **: statistically different from the wild type at 5%, 1% level, respectively.

*GhCYP724B* was predicted to be a target gene of miR3367 (a newly detected miRNA in cotton) (Figures 2A, S7A). Using rapid amplification of complementary DNA (cDNA) 5′ ends (5′‐RACE) assays, we confirmed the cleavage of *GhCYP724B* transcripts directed by miR3367 *in vivo* (Figures 2B, S7B). lncRNA67 paired with miR3367, with almost perfect complementarity, except for a three‐base bulge at nucleotides 9–11 at the 5′ end (Figure 2C). To validate the direct binding between miR3367 and lncRNA67, we performed a microscale thermophoresis assay using labeled lncRNA67 and different concentrations of miR3367. The lncRNA67 had concentration‐dependent effects on fluorescently labeled miR3367, with a *K*_*d*_ value of 2.6535E–07 M (Figure 2D), indicating the specific binding of lncRNA67 and miR3367.

To determine whether lncRNA67 acts as an eTM of miR3367, we constructed overexpression vectors containing *lncRNA67* (OE‐lncRNA67), a mutated lncRNA67 named *mlncRNA67* (OE‐mlncRNA67), and *miR3367* (OE‐Pre‐miR3367), respectively. We used these vectors to transiently express these RNAs in *Nicotiana benthamiana* leaves. Co‐expression of the miR3367 precursor and its target (*35S:GhCYP724B‐GFP*) led to a reduction in green fluorescent protein (GFP) fluorescence, suggesting that miR3367 mediates the degradation of *GhCYP724B* (Figure 2E). The GFP fluorescence intensity was recovered in the presence of OE‐lncRNA67, but not OE‐mlncRNA67, indicating that the miR3367‐mediated cleavage of its target *GhCYP724B* was blocked by the overexpression of *lncRNA67* (Figure 2E). Notably, the levels of miR3367 were much higher in *lncRNA67* knockout mutants (LM04 and LM13) than in the WT and *GhCYP724B* mutants (GM07 and GM19) (Figure 2F), supporting the notion that lncRNA67 binds to miR3367 and inhibits the miR3367‐mediated cleavage of *GhCYP724B*. These results indicate that lncRNA67 positively regulates the expression of *GhCYP724B* by acting as an eTM of miR3367.

### Long non‐coding RNA67 and *GhCYP724B* positively regulate male sterility

**Figure 3:**
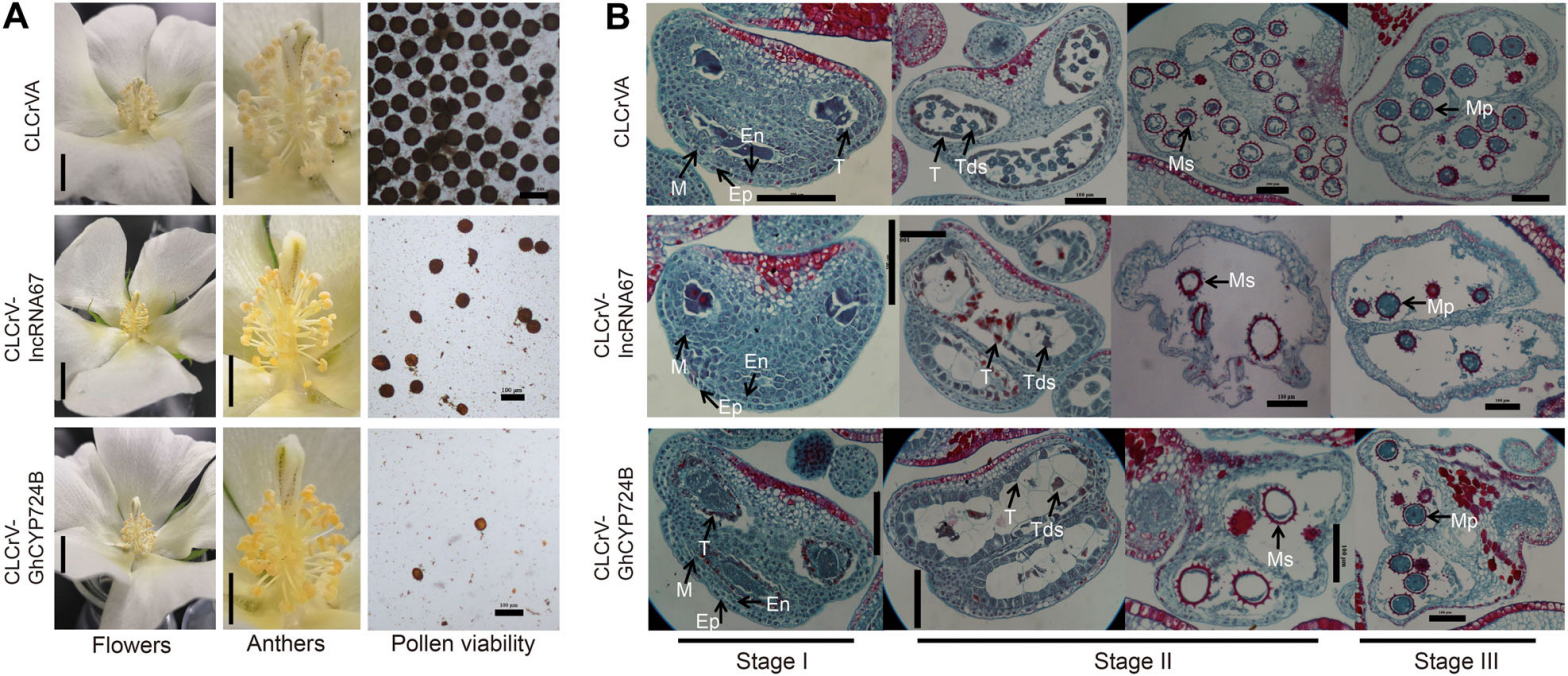
**Silencing** ***lncRNA67*** **and** ***GhCYP724B*** **resulted in male sterility in upland cotton** **(A)** Flowers, anthers, and pollen viability detection of cotton plants silenced long non‐coding RNA67 (Cotton leaf crumple virus (CLCrV)–lncRNA67), *Gossypium hirsutum* cytochrome P724B (CLCrV–GhCYP724B), and control (CLCrVA). Pollen viability was detected by iodine – potassium iodide (I_2_‐KI) staining method. Scales bars in the chart of flowers and anthers, 1 cm. Scales bars in the chart of pollen viability detection, 100 μm. **(B)** Comparison of developing anthers and microspores among gene‐silenced plants (CLCrV–GhCYP724B, CLCrV–lncRNA67), and the control. En, endothecium; Ep, epidermis; M, middle layer; Mp, mature pollen; Ms, microspore; T, tapetum; Tds, tetrads. Stage I: flower bud < 1.5 mm; Stage II: 1.5 mm ≤ flower bud ≤ 8.0 mm. Stage III: flower bud >8.0 mm. Scales bars, 100 μm.

To assess the potential functional impact of lncRNAs during pollen development, we silenced 10 lncRNAs in cotton lines 2074A and 2074B. Silencing *GhChlI* caused a photobleaching phenotype in cotton leaves, and its target gene was silenced by approximately 60% (Figure S8). When *lncRNA67* was silenced in 2074B (Cotton leaf crumple virus (CLCrV)–*lncRNA67*), the anthers were withered, and no pollen grains were released during flowering (Figure 3A). Iodine (I_2_) – potassium iodide (KI) staining showed that few pollen grains were produced by the CLCrV–lncRNA67 plants, and the grains that were produced were irregular in shape and light in color (Figure 3A). lncRNA67 targets 13 protein‐coding genes involved in binding, signal transduction, and secondary metabolite biosynthetic processes in cotton (Figures S9, S10A). The transcript abundance of *GhCYP724B*, a target of lncRNA67, was significantly reduced in the flower buds of CLCrV–lncRNA67 compared to the controls (CLCrVA and 2074B) (Figure S10B). Gh*CYP724B* and *lncRNA67* had similar expression patterns in different plant species, and both were highly expressed in flower buds of fertile lines (Figure S10C).

A subcellular localization analysis indicated that GhCYP724B was localized on the plasma membrane of *N. benthamiana* epidermal cells (Figure S10D). Additionally, *GhCYP724B*, which is involved in BR biosynthesis, interacts with *lncRNA67* (Figure 1G). *GhCYP724B*‐silenced 2074B plants (CLCrV–*GhCYP724B*) exhibited male‐sterile phenotypes, with fewer activated pollen grains, like *lncRNA67*‐silenced 2074B plants (CLCrV–*lncRNA67*) (Figure 3A). A cytological observation of anthers in paraffin sections showed no difference at the sporogonium stage (stage I) among CLCrV–*lncRNA67*, CLCrV–*GhCYP724B*, and WT plants (Figure 3B). The tapetum cells started to degenerate, and larger tetrads formed in anthers during the stage from pollen mother cell to late UNP (stage II) in the WT (Figure 3B); however, the microsporocytes in CLCrV–*lncRNA67* and CLCrV–*GhCYP724B* plants did not further develop into tetrads, and the cells began to die, resulting in fewer mature pollen grains (Figure 3B). This phenomenon is analogous to the phenotypes of BR‐biosynthesis and signaling mutants in Arabidopsis (Ye et al., 2010), suggesting that defects in BR biosynthesis result in male sterility across a variety of plant species. These results demonstrate that BR biosynthesis was blocked after the knockdown or knockout of *lncRNA67* and *GhCYP724B* in upland cotton.

**Figure 4:**
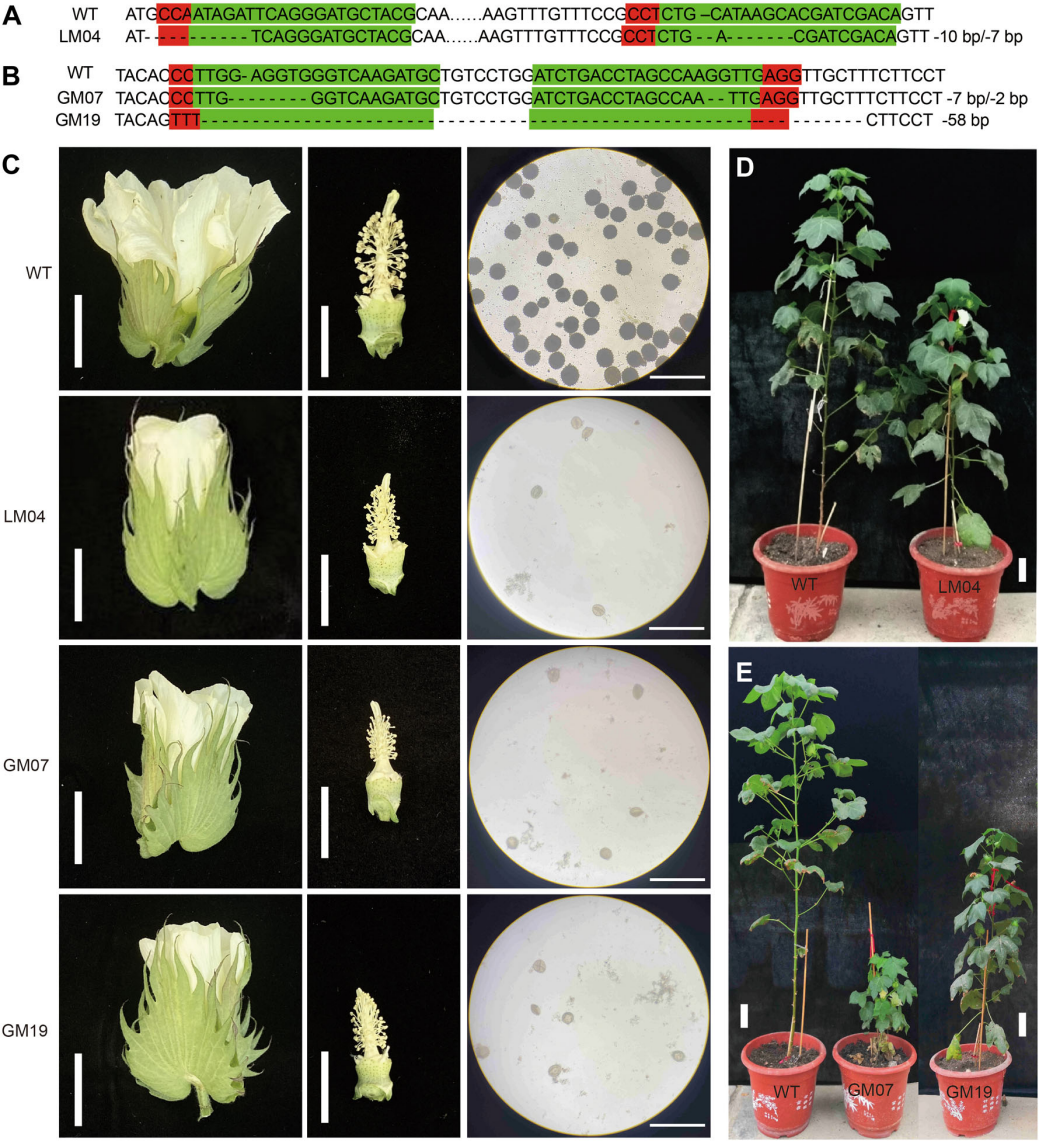
**The knockout of** ***lncRNA67*** **and** ***GhCYP724B*** **in upland cotton leads to male sterility and dwarfism phenotypes** **(A)** Sanger sequencing analysis of genome editing at single‐guide RNA1 (sgRNA1) and sgRNA2 sites of *long*‐*non*‐*coding* *RNA67* (*lncRNA67*) in *KO‐lncRNA67* plants. **(B)** Sanger sequencing analysis of genome editing at sgRNA1 and sgRNA2 sites of *Gossypium hirsutum* cytochrome P724B (*GhCYP724B*) in *KO‐GhCYP724B* plants. The sgRNA target sites and the protospacer adjacent motif (PAM) regions are highlighted in green and red background, respectively. Nucleotide deletions or insertions are labeled at right. **(C)** Flowers and pollen activity detection in *GhCYP724B* mutants, *lncRNA67* mutant and WT. GM, *GhCYP724B* mutants; LM, *lncRNA67* mutants; WT, the wild type. Scale bars in the flowers, 2 cm. Scale bars in the photo of pollen activity detection, 500 μm. Phenotype of *lncRNA67* mutants **(D)** and *GhCYP724B* mutants **(E)**, Scale bars, 10 cm.

To further investigate the functions of lncRNA67 and *GhCYP724B*, we generated 20 knockout mutants of *GhCYP724B* and 15 knockout mutants of *lncRNA67* in upland cotton via CRISPR/Cas9‐mediated gene editing (Figure S11A, B). Five *GhCYP724B* mutants (GM07, GM19, GM18, GM24, and GM29) and three *lncRNA67* mutants (LM04, LM12, and LM13) were male sterile (Figure S11C). Three *GhCYP724B* mutants (GM07, GM18, and GM19) and one *lncRNA67* mutant (LM04) were subjected to further analysis (Figure 4A–E). The LM04 mutant had an 11‐bp deletion in the single‐guide RNA1 (sgRNA1) locus (Figure 4A). The *GhCYP724B* sequences in GM07 and GM19 showed a 7‐bp deletion causing a frame shift and a 58‐bp deletion causing early termination, respectively (Figure 4B). In addition, these *GhCYP724B* mutants showed no off‐target mutations (Figure S12).

Under natural conditions in the greenhouse, the flowers of LM04 and LM13 showed similar male‐sterile characteristics to CLCrV–*lncRNA67* flowers, such as withered stamens, irregular pollen grains, and a light color after I_2_‐KI staining (Figures 4C, S11). In addition, these mutant plants were shorter than the WT at the vegetative and reproductive stages (Figures 4D, S13). Notably, the CRISPR/Cas9‐induced *GhCYP724B* mutants showed similar male‐sterile and dwarf phenotypes (Figures 4C, E, S11). We repeated the experiment in the field in the summer of 2023 and observed similar phenotypes (male sterility and dwarfism) in GM08 (a mutant of *GhCYP724B*) (Figure S14). These results indicate that the knockout of *lncRNA67* and *GhCYP724B* led to symptoms of BR deficiency, male sterility, and dwarfism.

### Knockdown of *CYP724B* in *N. benthamiana* results in symptoms of BR deficiency

*GhCYP724B* is a homolog of Arabidopsis *AtDWF4* (Figure S15). Phylogenetic analysis revealed that *GhCYP724B* clustered with *NtCYP724B1‐like* from tobacco (*N. tabacum*), *TaCYP* from wheat (*Triticum aestivum*), and *OsCYP724B1‐like* from rice (Figure S16). Knockdown of the homolog of *NtCYP724B1‐like* in *N. benthamiana* using RNAi resulted in dwarfism and leaf curling in the resulting RNAi‐*CYP724B* plants (Figure S17A). The expression level of *NbCYP724B* was significantly reduced in RNAi‐*CYP724B* plants compared to the control (Figure S17B). I_2_‐KI staining showed that some of the pollen grains in the transgenic *N. benthamiana* plants had irregular shapes and a light color (Figure S17C). Although flower bud length and width did not change markedly in these plants (Figure S17E), the number of seeds per boll was significantly reduced compared to the control (Figure S17D, E). The 1,000‐grain weights of RNAi‐*CYP724B*‐3 and RNAi‐*CYP724B*‐5 were higher than that of the control (Figure S17F), but their seed germination rates showed no significant differences (Figure S17G, H). In conclusion, silencing *NbCYP724B* in *N. benthamiana* evoked symptoms of BR deficiency and male semi‐sterility.

### *GhCYP724B* regulates pollen development by affecting BR homeostasis

**Figure 5:**
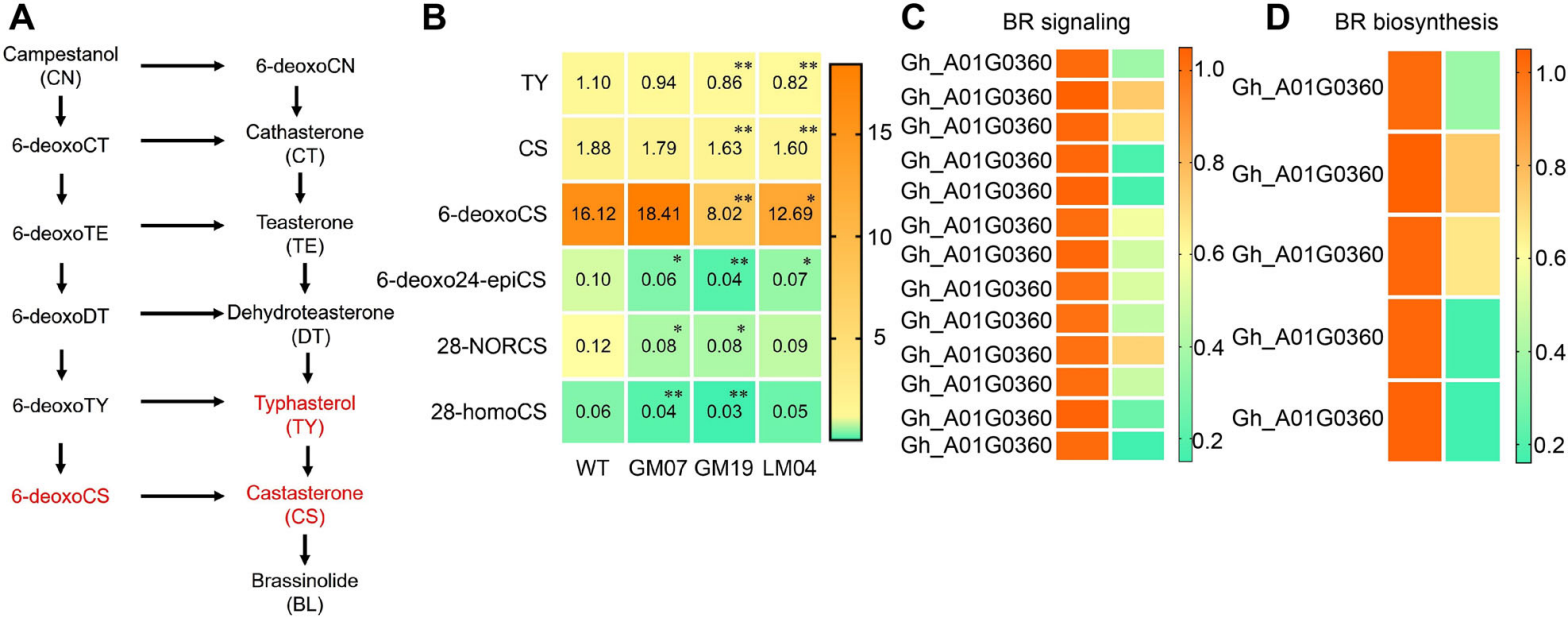
**The knockout** ***GhCYP724B*** **in upland cotton inhibits bassinosteroid (BR) biosynthesis and BR signaling genes expression** **(A)** The diagram of BR biosynthesis pathway. **(B)** Endogenous BR contents in the flower buds of long non‐coding RNA67 (lncRNA67) mutants, *Gossypium hirsutum* cytochrome P724B (GhCYP724B) mutants and wild type (WT) plants. 6‐DeoxoCS, 6‐deoxocastasterone; 6‐deoxo‐24‐epiCS, 6‐deoxo‐24‐epicastasterone; 28‐homoCS, 28‐homocastasterone; 28‐norCS, 28‐norcastasterone; CS, castasterone; TY, typhasterol. *, **: statistically different from the WT at 5% and 1% levels, respectively. Expression profiles of genes in BR signaling **(C)** and BR biosynthesis **(D)** in the flower bud of *GhCYP724B* mutant GM19 and Jin668.

*GhCYP724B* encodes a 22α hydroxylase that controls a rate‐limiting step in the BR biosynthetic pathway. To further explore the roles of lncRNA67 and GhCYP724B in BR biosynthesis, we analyzed the endogenous BR contents throughout pollen development using ultra‐performance liquid chromatography – tandem mass spectrometry (UPLC–MS/MS). The levels of 28‐homocastasterone (28‐homoCS), 28‐norcastasterone (28‐norCS), 6‐deoxocastasterone (6‐deoxoCS), typhasterol (TY), and castasterone (CS) were lower in the flower buds of the *lncRNA67* and *GhCYP724B* mutants than the WT (Figure 5A, B; Table S8). In addition, six BR‐signaling genes and nine BR‐biosynthesis genes were significantly downregulated in the *GhCYP724B* mutant compared to the WT (Figure 5C, D).

**Figure 6:**
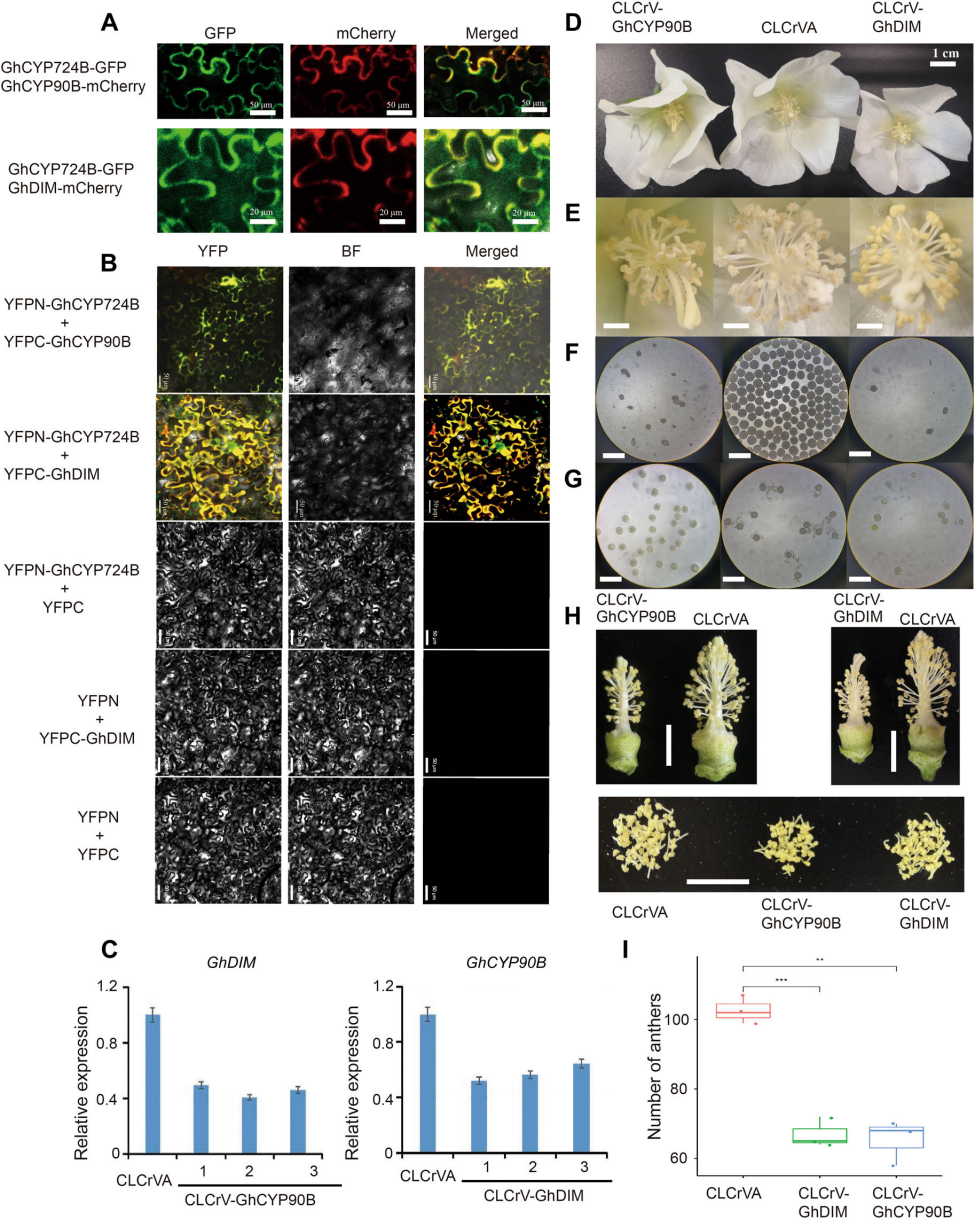
**Silencing of** ***GhDIM*** **and** ***GhCYP90B*** **affected the male fertility in upland cotton** **(A)** Co‐localization analysis between *Gossypium hirsutum* cytochrome P724B (GhCYP724B) and two interacted proteins in the epidermal cells of tobacco leaves. Scale bars, 20 μm. **(B)** Bimolecular fluorescence complementation (BiFC) assay identification the interaction between GhCYP724B and GhCYP90B/GhDIM. Scale bars, 50 μm. **(C)** Expression pattern of *GhDIM* and *GhCYP90B* in virus‐induced gene silencing (VIGS) plants. **(D)** The flower of VIGS plants. **(E)** Anthers from the VIGS transgenic plants and control plants. **(F)** Pollen viability detection by iodine – potassium iodide (I_2_‐KI) staining method for VIGS transgenic and control plants. Scale bars, 200 μm. **(G)** The germination of pollen grains of VIGS transgenic and control plants. Scale bars, 200 μm. **(H)** Comparison of the anther status of VIGS and control plants. Scale bars, 1 cm. **(I)** The number of anthers of VIGS and control plants.

Previous studies have shown that CYP724B interacts with DIM, DET2, CYP90B, and ROT3 in Arabidopsis (Figures S18). *GhDIM* encodes a delta (24)‐sterol reductase, and *GhCYP90B* encodes a member of the cytochrome P450 protein family, both of which are involved in BR biosynthesis. GhCYP90B, GhDIM, and GhCYP724B localized to the plasma membrane in *N. benthamiana* leaves co‐expressing these proteins (Figure 6A). We validated the interactions of these proteins with GhCYP724B using a bimolecular fluorescence complementation (BiFC) assay. When *YFPN–GhCYP724B* was co‐expressed with *YFPC–GhDIM* or *YFPC–GhCYP90B* in the epidermal cells of *N. benthamiana* leaves, yellow fluorescent signals were detected on the cell membrane (Figure 6B), indicating that GhCYP724B physically interacts with GhDIM and GhCYP90B *in vivo*. These data suggest that GhCYP724B regulates BR metabolism by interacting with GhDIM and GhCYP90B. Furthermore, silencing *GhDIM* and *GhCYP90B* in upland cotton line 2074B resulted in sterility or semi‐sterility (Figure 6C–E). The viability and germination rates of the pollen grains and the number of anthers were reduced in these transgenic plants (Figure 6F–I). These results indicate that GhDIM and GhCYP90B positively regulate pollen fertility in upland cotton.

### Brassinosteroids control male sterility by regulating the genes involved in pollen development

**Figure 7:**
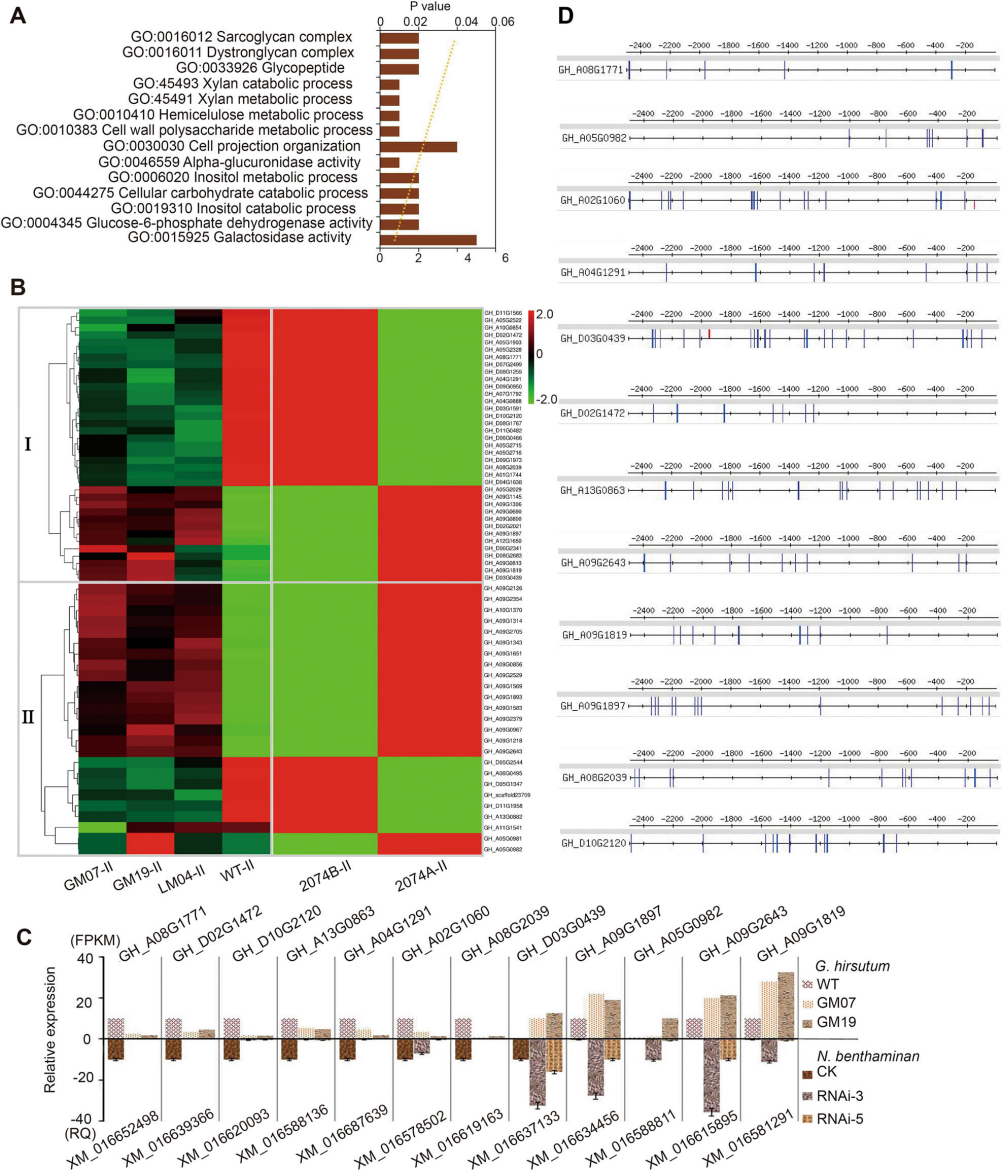
*BES1*/*BZR1* directly binds to the promoter regions of key genes and regulates male sterility **(A)** Gene Ontology annotation and cluster categories of differentially expressed genes (DEGs) co‐existed in GM07‐F versus WT‐F, GM19‐F versus WT‐F, and LM04‐F versus WT‐F comparisons. **(B)** Heat map of expression profiles for co‐existed DEGs across different male sterile and fertile lines; GM19‐F, GM07‐F, LM04‐F, and WT‐F indicate the flower buds at the pollen mother cells to late uninucleate pollen (UNP) stage in clustered regularly interspaced short palindromic repeats/CRISPR‐associated protein 9 (Cas9) transgenic plants and wild type (WT) plants. 2074A‐II and 2074B‐II, see legend in Figure 3. **(C)** Genes related to pollen development showed similar expression trends after silencing the *CYP724B* in cotton and tobacco. The expression abundance of cotton genes was evaluated by RNA sequencing, and their homologs in tobacco were detected by reverse transcription – quantitative polymerase chain reaction (RT‐qPCR). **(D)** Promoter regions of genes in regulating pollen development contain DNA elements of E‐box (CANNTG) and BRREs (CGTGT/CG). Blue bars indicate E‐boxes and red bars indicate BRREs. Two‐thousand and five hundred base pair upstream sequences from the start codon of each gene were obtained from the TM‐1 genome and analyzed using the “Regulatory Sequence Analysis Tools” (http://rsat.ulb.ac.be/rsat/).

By comparing the genes expressed in flower buds at the stage from pollen mother cell to late UNP formation between the *GhCYP724B* mutants, *lncRNA67* mutants, and the WT, we identified 848 common differentially expressed genes (DEGs) in the GM07‐F versus WT‐F, GM19‐F versus WT‐F, and LM04‐F versus WT‐F comparisons (Figure S19A; Table S9). Among these, 127 DEGs are involved in carbohydrate, lipid, and fatty acid metabolism, with functions in processes such as cell wall polysaccharide metabolic processes, xylan catabolic processes, and lipid biosynthetic processes (Figures 7A, S19B). These metabolic processes are critical for tapetum and microspore development (Corral‐Martínez et al., 2019). Additionally, some DEGs are involved in plant hormone signaling pathways (Figure S19C) and might regulate pollen development via a hormone‐dependent pathway. Notably, 37 of the 127 DEGs were differentially expressed not only between the CRISPR/Cas9 mutants and the WT, but also between 2074A and 2074B (Figure 7B).

We examined the expression patterns of the homologs of 12 randomly selected DEGs in *N. benthamiana*, which revealed similar trends to the cotton plants in the *NbCYP724B‐*silenced *N. benthamiana* (Figure 7C). Based on these results, we predict the existence of a common transcription factor that regulates the expression of genes associated with pollen development in both cotton and tobacco. The transcription factor BES1/BZR1 specifically regulates BR signal transduction. Dephosphorylated BES1/BZR1 targets thousands of downstream BR‐responsive genes by binding to the cis‐elements in their promoters, such as E‐boxes and BR response elements. Indeed, the promoter regions of the DEGs associated with tapetum and microspore development contain multiple binding sites that could be targeted by BES1/BZR1 (Figures 7D, S20). These findings suggest that BRs control male sterility by regulating the expression of genes involved in pollen development.

### The mitochondrion‐specific ORF *Aorf27* contributes to the CMS of 2074A

**Figure 8:**
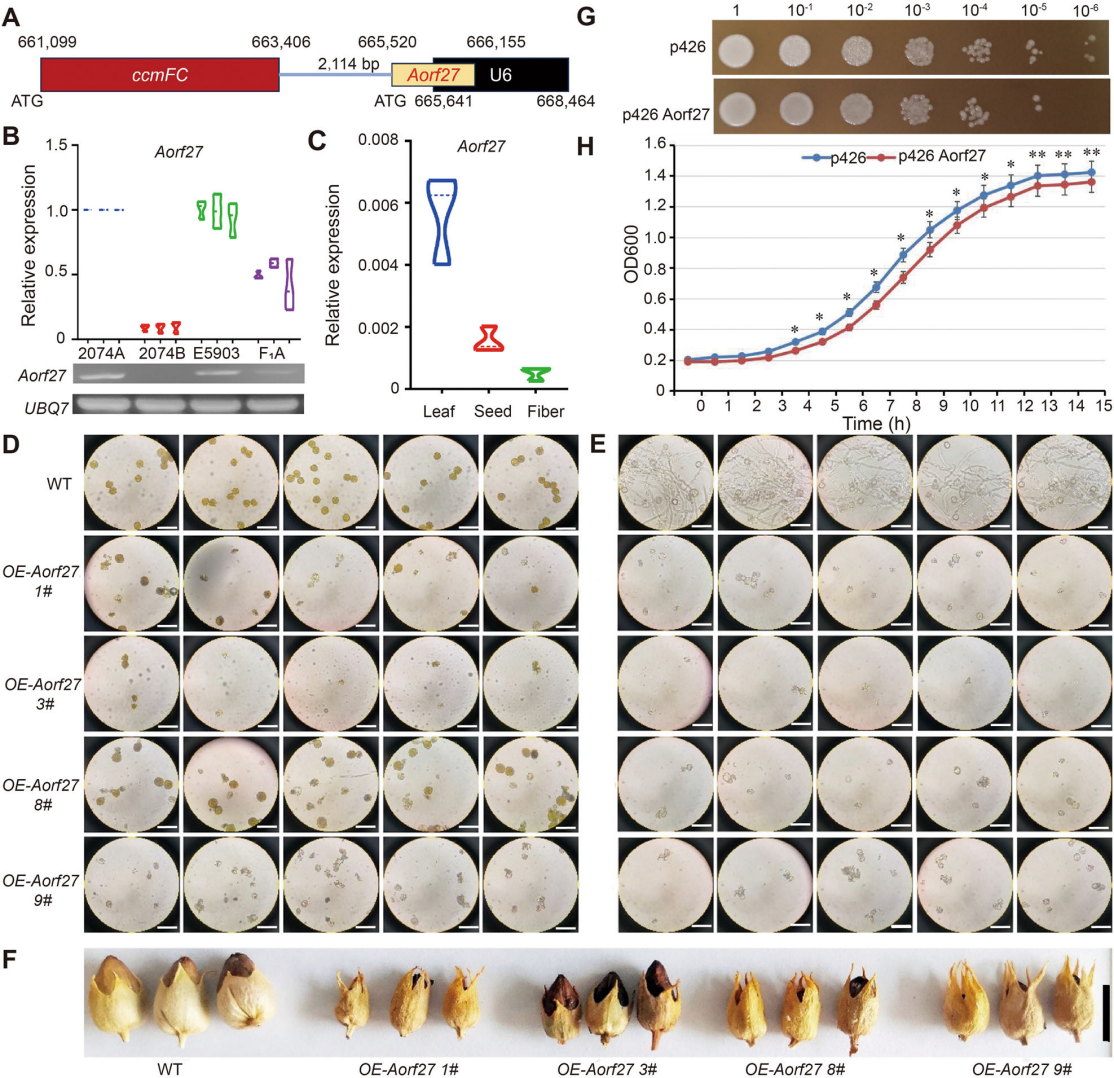
**Over expression of** ***Aorf27*** **caused male sterility in tobacco and was toxic to yeast** **(A)** Genomic structures of cytoplasmic male sterility‐associated open reading frames in upland cotton. **(B)** Expression patterns of *Aorf27* in 2074A, 2074B, E5903 and F_1_‐A floral buds (1.5–8.0 mm). Three primer pairs were designed from the coding sequences (CDS）region of *Aorf27*. The gel chart on the bottom is the semi‐quantitative polymerase chain reaction analysis of *Aorf27* in 2074A, 2074B, E5903, and F_1_‐A. E5903, the restorer line; F_1_‐A, three‐line hybrid. **(C)** Expression pattern of *Aorf27* in the leaf, seed, and fiber of TM‐1. **(D)** Iodine – potassium iodide (I_2_‐KI) staining of pollen. Two anthers per flower were used in the I_2_‐KI staining. Each field of view is an image magnified by 400 under a light microscope. Scale bars, 1 mm. **(E)** Germination experiment of pollen. Each field of view is an image magnified by 400 under the light microscope. Scale bars, 1 mm. **(F)** Pod phenotype of tobacco positive transformants. Scale bars, 1 cm. The seed pods of 3# were obtained with wild type (WT)‐assisted pollination in Figure 8F. **(G)** Growth of transformed yeast cells. Yeast cells with p426 and p426Aorf27 plasmids were cultured in liquid buffer C (0.15 mol/L NaCl, 10 mmol/L Bicine, pH: 8.35) at 30°C 1 h. The cultures were then diluted 1, 10, 100, 1,000 fold with liquid buffer C, after which each serial dilution was dripped on synthetic complete –Uracil (SC‐ura) agar plates. The yeast cells were cultured at 30°C for 3 d. **(H)** Growth curve of transformed yeast cells in liquid medium. The density of the transformed yeast cells was determined every hour by measuring the optical density at 600 nm. Cells were cultured for 15 h in SC‐ura liquid media at 30°C.

Cytoplasmic male sterility is affected by MT genes or mitochondrial ORFs (Chen et al., 2017). We previously compared all mitochondrial ORFs between 2074A and 2074B and identified 28 novel ORFs in 2074A (*Aorf1* to *Aorf28*) (Li et al., 2018a). Here, we determined that some of the 848 DEGs detected between the CRISPR/Cas9 mutants GM07/GM19//LM04 and WT flower buds not only interact with the novel ORFs (*Aorf25* and *Aorf27*), either directly or indirectly (Figure S21A), but also with MT genes. *GhCYP724B* was predicted to be co‐expressed with the MT genes *cox3*, *ccmFN*, *rps7*, *nad7*, and *atp8* (Figure S21B). Additionally, GhCYP724B not only directly interacts with GH_A09G1819, but also indirectly interacts or is co‐expressed with *Aorf25* and *Aorf27* (Figure S21C). The genes involved in this network showed differential expression patterns between the sterile and fertile lines (Figure S21D). *Aorf27* is located adjacent to *ccmFC* in the unique region of the sterile line 2074A (Figure 8A). *Aorf27* was expressed at high levels in 2074A and the hybrid F_1_A, but its expression was almost undetectable in the maintainer line 2074B (Figure 8B). In addition, *Aorf27* was barely expressed in the leaves, seeds, or fibers of upland cotton line TM‐1 (Figure 8C). *Aorf27* and *ccmFC* had similar expression trends in 2074B/2074A (Li et al., 2018a). By contrast, the expression levels of *Aorf25* showed no obvious differences among the four genetic materials (2074A, 2074B, the restorer line E5903, and the hybrid line F_1_‐A) but was highly expressed in the seeds and fibers of TM‐1 (Figure S21E, F).

To investigate the role of *Aorf27* in male sterility, we generated *Aorf27*‐overexpressing transgenic tobacco (*N. tabacum*) plants via Agrobacterium‐mediated transformation of leaf disks. We obtained 11 transformants, four of which (No. 1, No. 3, No. 8, and No. 9) were confirmed to be positive *Aorf27*‐overexpressing lines and showed phenotypic changes (Figure S22). Transformant No. 3 showed complete male sterility, forming irregularly shaped pollen grains that failed to normally germinate (Figure 8D, E). I_2_‐KI staining revealed that significantly fewer pollen grains were produced by transformant No. 1 compared to the WT (Figures 8E, S23A). The pollen germination rates of transformants No. 1, No. 8, and No. 9 were significantly lower than that of the WT (Figures 8E, S23B). The seed pods of the four transformants were significantly smaller than WT seed pods (Figure 8F). In addition, the seed pod weight, seed weight per pod, and number of seeds per pod were significantly reduced in the transformants (Table S10). These findings suggest that *Aorf27* might be responsible for the CMS of 2074A. In addition, the ectopic expression of *Aorf27* in yeast (*Saccharomyces cerevisiae*) reduced cell viability (Figure 8G) and growth rate (Figure 8H). In summary, *Aorf27* showed similar characteristics to CMS‐associated genes in other plants, and *GhCYP724B* might cooperate with *Aorf27* to facilitate CMS in 2074A upland cotton.

### GhCYP724B physically interacts with Aorf27

**Figure 9:**
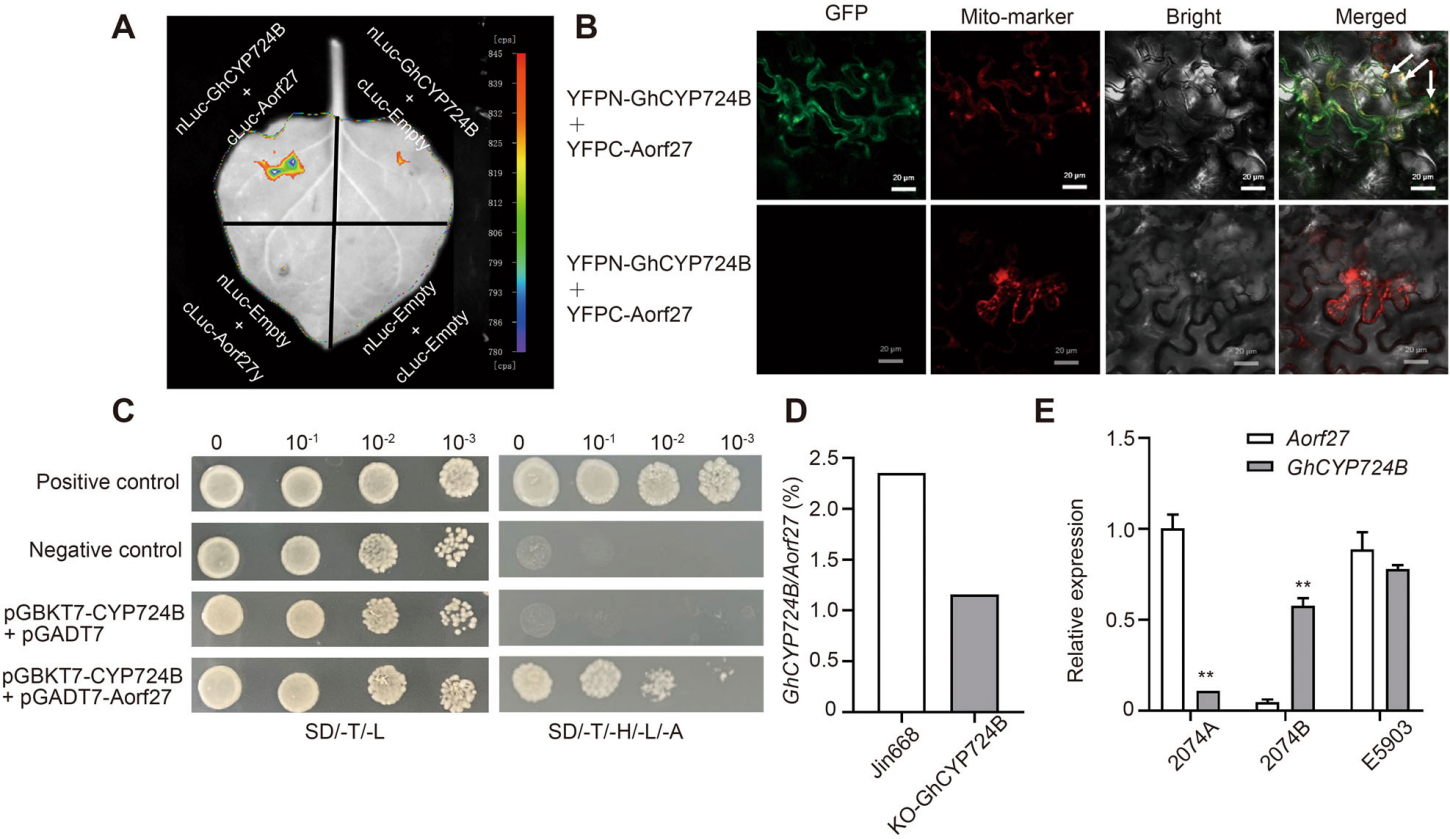
***Gossypium hirsutum*** **cytochrome P724B (GhCYP724B) physically interacts with the mitochondrion‐specific open reading frame Aorf27** **(A)** Luciferase complementation imaging (LCI) assays. The construct encoding nLUC‐tagged GhCYP724B was co‐infiltrated into the leaves of *Nicotiana benthamiana* plants along with a construct encoding cLUC‐targeted Aorf27. **(B)** Bimolecular fluorescence complementation (BiFC) assay showing that GhCYP724B interacts with Aorf27 in mitochondria. Constructs encoding GhCYP724B and Aorf27 fused to the N terminus or the C terminus of yellow fluorescent protein (YFP) were co‐infiltrated into the leaves of *N. benthamiana* plants. Scale bars, 20 μm. **(C)** Yeast two‐hybrid assay verified the interaction between GhCYP724B and Aorf27. **(D)** Expression patterns of *GhCYP724B/Aorf27* ratio in knockout (KO)‐GhCYP724B and Jin668, respectively. **(E)** Expression patterns of *GhCYP724B* and *Aorf27* in bud sof 2074A, 2074B, and E5903. * and ** indicate significant differences between *GhCYP724B* and *Aorf27* at the *P* = 0.05 and *P* = 0.01 levels, respectively.

To determine whether GhCYP724B interacts with Aorf27, we conducted multiple assays. In a luciferase complementation imaging (LCI) assay, co‐expression of cLUC‐GhCYP724B and nLUC‐Aorf27 in *N. benthamiana* leaves led to LUC activity (Figure 9A). Moreover, we confirmed the interaction between GhCYP724B and Aorf27 using BiFC assays, in which an excited yellow fluorescent protein (YFP) reconstituted signal was observed when YFPN–GhCYP724B and YFPC–Aorf27 were co‐infiltrated into *N. benthamiana* leaves (Figure 9B). In a yeast two‐hybrid (Y2H) assay, GhCYP724B interacted with Aorf27 in yeast cells (Figure 9C). Collectively, these results demonstrate that Aorf27 interacts with GhCYP724B.

We examined *GhCYP724B* and *Aorf27* transcript levels in KO‐GhCYP724B and Jin668 upland cotton using qRT‐PCR. We calculated the ratio of *GhCYP724B* to *Aorf27* expression to examine the relationship between the two genes. The *GhCYP724B/Aorf27* ratio was higher in Jin668 (2.35) than in KO‐GhCYP724B (1.16), indicating a negative correlation between *GhCYP724B* and *Aorf27* (Figure 9D). Finally, we examined the expression levels of *GhCYP724B* and *Aorf27* in the buds and sepals of the sterile line 2074A. When the expression level of *GhCYP724B* was reduced, the expression level of *Aorf27* significantly increased (Figure 9E). Based on these findings, we propose that GhCYP724B inhibits Aorf27 activity in a dose‐dependent manner.

## DISCUSSION

### The lncRNA–miRNA interaction influences male sterility in upland cotton

Male sterility systems enable the convenient and efficient production of cotton hybrids. The importance of lncRNAs for pollen development has been extensively described in various plant species (Lv et al., 2020). Based on previous studies (Li et al., 2014b; Wang et al., 2015; Cagirici et al., 2021; Nie et al., 2024), we adopted more stringent criteria to ensure the robust and reliable identification of candidate lncRNAs involved in pollen formation. (I) Unknown transcripts with a score <0 were retained in the datasets generated by three computational algorithms, Coding Potential Calculator (CPC; Kong et al., 2007), Coding‐Non‐Coding Index (CNCI; Sun et al., 2013), and Coding Potential Assessment Tool (CPAT; Wang et al., 2013. (II) To eliminate transcriptional noise and obtain reliable lncRNA datasets, transcripts with two or more exons were selected as lncRNA candidates (Kelley and Rinn, 2012; Pauli et al., 2012; Lv et al., 2013). (III) lncRNAs in which expression levels were higher than 0.1 in any sample were included. Based on the above standards, 3,855 reliable lncRNAs were retained for further analysis.

Long non‐coding RNAs interact with miRNAs and function as target mimics, an important regulatory mechanism for lncRNA activity during various biological processes (Ke et al., 2019). Here, we discovered that the lncRNA67–GhCYP724B interaction regulates male sterility in cotton by influencing BR metabolism. As an eTM of miR3367, lncRNA67 positively regulates the expression of *GhCYP724B*. In upland cotton, the precursor of miR3367 folded into a stem–loop structure and was transcribed in the flower buds of 2074A and 2074B (Figure S7). Therefore, we suggest that miR3367 is a novel miRNA in upland cotton that was likely not detected in a previous study (Nie et al., 2018) due to strict criteria for screening novel miRNAs.

### Long non‐coding RNA67 and GhCYP724B affect male sterility by regulating BR metabolism

Although studies on the mechanisms by which lncRNAs regulate reproductive development in plants lag far behind those in mammals, several lncRNAs were confirmed to play critical roles in flowering plants (Ding et al., 2012; Zhang et al., 2014; Fan et al., 2016). These lncRNAs show highly tissue‐specific expression patterns (Fatica and Bozzoni, 2013), especially in reproductive organs. In the current study, approximately 40% (123/307) of tissue‐specific lncRNAs were highly expressed in flower buds, which is consistent with findings in maize and rice (Zhang et al., 2014; Li et al., 2014b). We identified 63 lncRNAs that were not only expressed specifically in flower buds, but also differentially expressed between the CMS line and the maintainer line (Figure 1C), suggesting they play specific roles in male organ development and pollen fertility. Additionally, some of these 63 lncRNAs interact with BR‐related genes (Figure S6), indicating that lncRNAs related to BR metabolic processes are important for male sterility in cotton (Yu et al., 2019). Similarly, based on lncRNA sequencing during anther development in cotton CMS‐D_2‐2_ sterile line A and its maintainer line B, Zhang et al. (2019) also determined that lncRNAs that participate in phytohormone metabolism might also regulate male sterility.

Brassinosteroids play critical roles in regulating flowering, tapetal cell degeneration, and pollen development (Fujioka and Sakurai, 2003; Jiao et al., 2020; Yan et al., 2020). Endogenous BRs were discovered in the floral organs and pollen of *Brassica napus* (Grove et al., 1979), with CS and brassinolide (BL) showing significant biological activity in plants. In the present study, the levels of BR pathway intermediates were significantly reduced in the flower buds of the *GhCYP724B* mutants GM07 and GM19 and the *lncRNA67* mutant LM04 (Figure 5A).

Inhibiting BR biosynthesis or BR signal transduction decreases plant height and induces male sterility. In tomato (*Solanum lycopersicum*), SlSUT2 directly interacts with components of the BR‐signaling pathway, and *SlSUT2*‐knockdown plants showed a dwarf phenotype and male sterility (Hansch et al., 2020). Additionally, the basic helix‐loop‐helix (bHLH) protein BIM1 (BES1‐interacting Myc‐like 1) is a BR‐signaling component involved in regulating BR‐induced genes (Yin et al., 2005). The hypermethylation of *OsBIM2* affected male fertility in rice (Hu et al., 2015). Genetic studies in Arabidopsis indicated that the viability of pollen grains was reduced by 95%, 90%, and 94% in the BR‐biosynthesis mutant *cpd*, the BR‐perception mutant *bril‐116*, and the severe BR‐signaling mutant *bin2‐1*, respectively (Ye et al., 2010).

Knocking down *DWF4* in Arabidopsis resulted in symptoms of BR deficiency, such as severe dwarfism, dark round leaves, and sterility (Azpiroz et al., 1998). In the present study, silencing or knocking out *GhCYP724B* caused male sterility and dwarfing in upland cotton (Figures 3, 4), and BR‐biosynthesis and signaling genes were downregulated in these mutants (Figure 5C, D). *lncRNA67* was co‐expressed with *GhCYP724B*, encoding a key enzyme that catalyzes the rate‐limiting step of BR biosynthesis (Zhang et al., 2012; Ghosh, 2017). We observed BR deficiency symptoms during anther development in the *GhCYP724B* mutants (Tanaka et al., 2005; Ye et al., 2010). These results provide evidence that the lncRNA67–GhCYP724B module regulates male sterility by modulating BR biosynthesis. Additionally, GhCYP724B interacts with GhCYP90B and GhDIM, which are involved in BR biosynthesis. GhDIM is a delta (24)‐sterol reductase that catalyzes the conversion of 24‐methylenecholesterol to campesterol (Wang et al., 2018). The heterologous expression of *HrCYP90B1 Hippophae rhamnoides* in Arabidopsis resulted in a significant increase in BR content (Liu et al., 2020). In the present study, silencing *GhCYP90B* or *GhDIM* in cotton resulted in male sterility, which is similar to the phenotype of the *GhCYP724B* mutants (Figure 6D–I). These findings demonstrate the importance of maintaining normal BR biosynthesis and signal transduction for pollen fertility in cotton.

BES1/BZR1 is a key transcription factor which mediates BR signal transduction (Li et al., 2018b). BZR1/BES1 acts as an integrator or master regulator that directly interacts with key regulatory factors from other pathways during plant development (Li and He, 2020). We detected numerous DEGs in the flower bud transcriptomes of GM07‐F versus WT‐F, GM19‐F versus WT‐F, and LM04‐F versus WT‐F, many of which are involved in tapetum and microspore development (Figure 7B). Their homologous genes also showed similar patterns when we compared transgenic RNAi‐*CYP724B N. benthamiana* with the WT (Figure 7C). Remarkably, the promoter regions of the DEGs in flower buds typically contain multiple BES1/BZR1 binding sites (Figure 7D). We speculate that BRs regulate pollen development by binding to the promoters of genes downstream of BES1/BZR1.

### Model of miR2267–lncRNA67–GhCYP724B module regulating CMS

Cytoplasmic male sterility is attributed to the uncoordinated inheritance between MT genes/MT ORFs and nuclear genes (Chen and Liu, 2014). In the present study, 848 common DEGs were identified in the GM07‐F versus WT‐F, GM19‐F versus WT‐F, and LM04‐F versus WT‐F comparisons, some of which interact with MT genes and two novel ORFs (*Aorf25* and *Aorf27*) in 2074A (Figure S21). *Aorf27* was specifically expressed in flower buds of the sterile line 2074A to a much greater level than that in the maintainer line 2074B or the hybrid F_1_‐A (Figure 8B). The characteristics of *Aorf27* are similar to those of other CMS‐associated genes (Figure 8A), such as *T‐urf13* in maize (*Zea mays*) (Kennell and Pring, 1989), *orf224* in rapeseed (*B. napus*) (Wang et al., 2002), *orf256* in wheat (Liu et al., 2011), and *orf610a* in upland cotton (Zhang et al., 2022). When *Aorf27* was expressed in yeast, yeast growth was inhibited (Figure 8G, H), which is consistent with the effects of BT CMS ORF79 (Peng et al., 2009) and WA CMS WA352 in rice (Luo et al., 2013), S CMS ORF355 in maize (Xiao et al., 2020), and Nsa CMS ORF346 in rapeseed (Sang et al., 2021). *Aorf27* may therefore contribute to the induction of CMS alongside GhCYP724B in upland cotton. Furthermore, GhCYP724B physically interacts with Aorf27 (Figure 9). The *GhCYP724B*/*Aorf27* ratio in the KO‐GhCYP724B mutant (1.16) was lower than that in the WT (Jin668; 2.35), indicating that when the content of GhCYP724B decreased, its suppression of *Aorf27* was weakened, resulting in the recovery of fertility.

**Figure 10:**
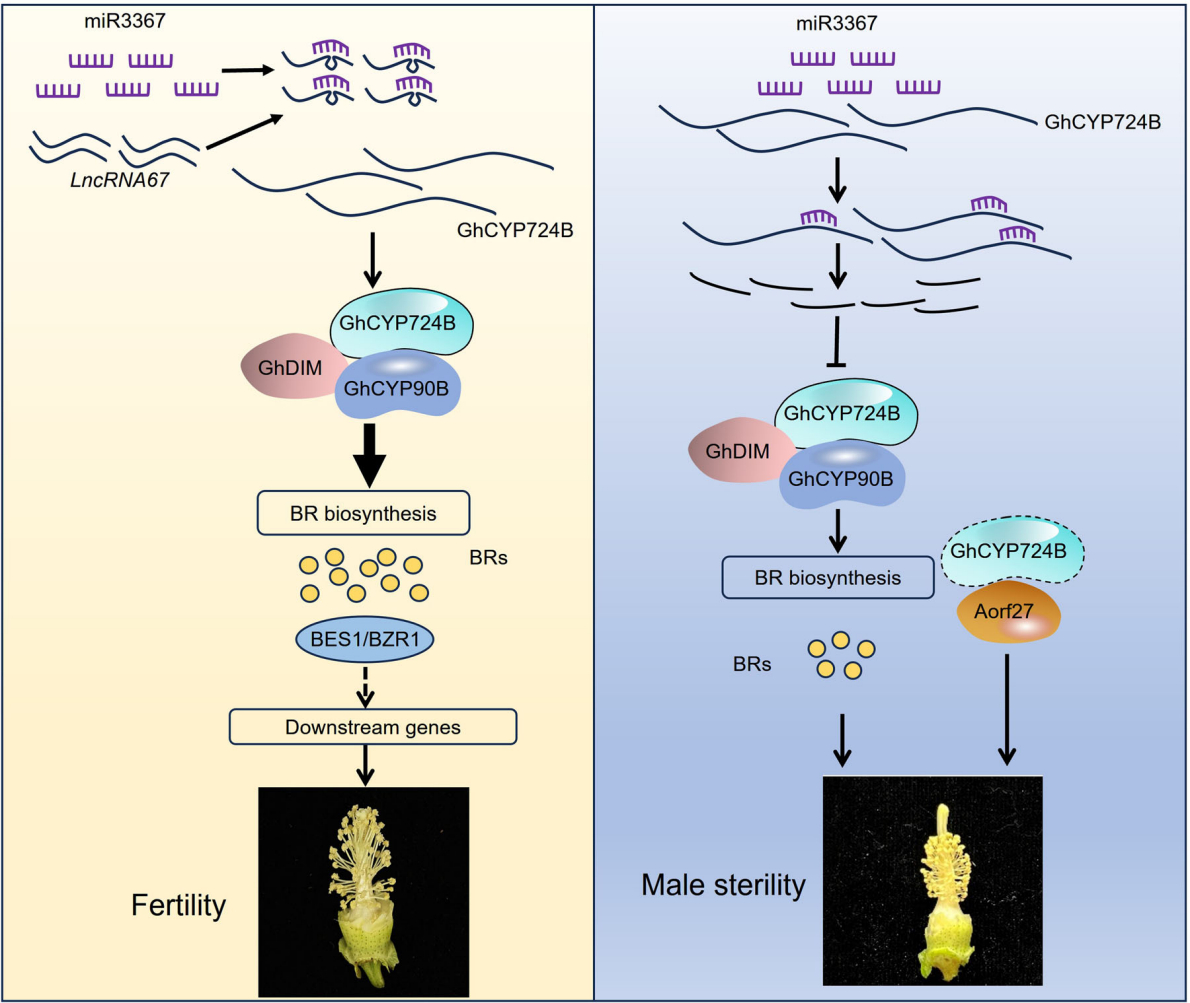
**The working model of microR3367 – long‐non‐coding RNA67 –** ***Gossypium hirsutum*** **cytochrome P724B (miR3367–lncRNA67–GhCYP724B) module regulates male sterility by modulating brassinosteroid (BR) synthesis in upland cotton** In maintained line 2074B, *lncRNA67* positively regulates *GhCYP724B* that function as eTMs (endogenous target mimics) by blocking the cleavage of GhCYP724B by miR3367, and induced the biosynthesis of BRs. BRs induce the expression of fertility related genes, which promote cotton fertility. However, in sterile line 2074A, the disruption of *lncRNA67* resulted in the suppression of GhCYP724B, then downregulated the expression of BR synthesis genes. The reduction of BR biosynthesis resulted in abnormal microspores development and apical meristem differentiates. GhCYP724B suppressed the expression of the specific *orf* (*Aorf27*) in 2074A, which led to toxic effects on cells. When the lncRNA67–GhCYP724B module was inhibited, Aorf27 up‐regulated expression will result in cotton male sterility.

In summary, the miR3367–lncRNA67–GhCYP724B module influences BR biosynthesis, thereby regulating male sterility (Figure 10). In the maintainer line 2074B, *lncRNA67* is highly expressed, which efficiently sequesters a large amount of miR3367, thereby suppressing the miR3367‐mediated cleavage of *GhCYP724B*. Thus, GhCYP724B induces BR biosynthesis by forming a complex with GhCYP90B and GhDIM, which results in an increase in BR content and fertile pollen production. In the CMS line 2074A, miR3367 binds to its target *GhCYP724B* and inhibits *GhCYP724B*‐mediated BR biosynthesis. The reduced BR content results in abnormal microspore development. The low expression of *GhCYP724B* weakens its suppression of *Aorf27*, an ORF specifically expressed in 2074A, which further induces the abortion of the pollen grains (Figure 10). Additionally, BES1/BZR1 may regulate plant height by controlling the development of the apical meristem and the nutrient supply by affecting downstream target gene expression.

Our findings on the interactions between GhCYP724B and genes involved in the BR‐signaling pathways shed new light on the regulatory network that coordinates male sterility and plant height. These insights could facilitate the genetic modification of CMS in upland cotton. Moreover, our findings provide materials for three‐line hybrid breeding for cotton seed production.

## MATERIALS AND METHODS

### Plant materials

The CMS line 2074A contains CMS‐D_2‐2_ cytoplasm, and the maintainer line 2074B (*G. hirsutum*) contains normal male‐fertile CMS‐AD_1_ cytoplasm (Li et al., 2013). E5903 is a restorer line of 2074A. The F_1_‐A hybrid was generated by crossing 2074A with E5903.

Pollen development in 2074A and 2074B was histologically analyzed at various developmental stages based on the size (length) of flower buds. The paraffin sections were stained with 1% safranine T and 1% fast green FCF (Liu et al., 2015) and imaged under an optical microscope (Olympus BX51; Olympus, Tokyo, Japan).

Tobacco (*N. tabacum* cv. Xanthi‐nc) seeds were sown in half‐strength Murashige and Skoog (MS) medium and cultivated in a controlled chamber at 28°C under a 16/8 h day/night cycle. Sterile young tobacco leaves were used for leaf disk transformation.

### Measurement of nutrient contents and antioxidant enzyme activities

The nutrient contents and malondialdehyde levels in flower buds were measured according to Cakmak and Horst (1991). Antioxidant enzyme (SOD, POD, and CAT) activities were measured as previously described (Xu et al., 2019). All experiments were run in triplicate, including biological and technical replicates, and datasets were calculated using mean ± *SE* values.

### Library preparation and sequencing

Stamens were isolated from flower buds at the sporogonium stage (stage I: flower bud < 1.5 mm) and pollen mother cells at the UNP stage (stage II: 1.5 mm < flower bud < 8.0 mm) for lncRNA sequencing. Four samples (2074A‐I, 2074A‐II, 2074B‐I, and 2074B‐II) were examined, with three biological replicates. Each ~1.5 μg RNA sample was subjected to ribosomal RNA (rRNA) removal using a Ribo‐Zero rRNA Removal Kit (Illumina, San Diego, CA, USA). Sequencing libraries were generated using a NEBNext Ultra Directional RNA Library Prep Kit (New England Biolabs, Ipswich, MA, USA) following the manufacturer's recommendations, and index codes were added to attribute sequences to different samples. The index‐coded samples were clustered with the acBot Cluster Generation System using a TruSeq PE Cluster Kit v3‐cBot‐HS (Illumina) according to the manufacturer's instructions. Following cluster generation, the lncRNA libraries were sequenced on the Illumina HiSeq platform, and paired‐end reads were generated.

### Quality control and lncRNA analysis

Transcripts were assembled using Cufflinks and Scripture based on reads mapped to the *G. hirsutum* accession TM‐1 reference genome (Zhang et al., 2015). The assembled transcripts were annotated using the Cuffcompare program, and novel transcripts were used to identify putative lncRNAs (Trapnell et al., 2012). Three computational approaches, CPC (Kong et al., 2007), CNCI (Sun et al., 2013), and CPAT (Wang et al., 2013), were combined to sort non‐protein‐coding RNA candidates from putative protein‐coding RNAs in the novel transcripts. Non‐protein‐coding transcripts >200 nt long with more than two exons were selected as lncRNA candidates. lncRNA transcripts with fragments per kilobase of exon per million mapped fragments (FPKM) ≤0.1 were also eliminated.

### Tissue specificity and differential expression analysis

The public Illumina RNA‐seq data from 10 tissues (leaves, petals, 10 d post‐anthesis (DPA) fibers, 20 DPA fibers, 10 DPA seeds, 20 DPA seeds, 30 DPA seeds, 40 DPA seeds, 0 DPA ovules, 30 DPA ovules) and two samples (2074B‐I flower buds, 2074B‐I flower buds) were used to analyze tissue specificity. The tissue specificity of the lncRNAs was evaluated according to the TSI, as previously described (Hao et al., 2015). The TSI was calculated based on the FPKM of each lncRNA in different tissues. LncRNAs with TSI > 0.85 were defined as tissue‐specific lncRNAs. The differential expression levels of lncRNAs between two samples were analyzed using Cuffdiff software (Trapnell et al., 2012). Only lncRNAs with an absolute log_2_ ratio ≥1 and a false discovery rate (FDR) significance score <0.05 were considered to be differentially expressed lncRNAs.

### Targeted gene prediction and functional enrichment analysis

Protein‐coding genes within 50 kb upstream or downstream of lncRNAs are subjected to cis‐regulation by lncRNAs (Yan et al., 2017). The trans‐acting lncRNAs and their target genes were predicted using LncTar software (Li et al., 2014a). GO analysis was carried out using the Web Gene Ontology Annotation Plot (http://wego.genomics.org.cn/). The protein–protein interaction networks were predicted using the STRING website (https://cn.string‐db.org/cgi/input.pl).

### Quantitative RT‐PCR and semi‐quantitative RT‐PCR

RNA was extracted from roots, stems, leaves, and flower buds using the improved cetyltripethylammonium bromide method (Cheng et al., 2023). The miRNAs were reverse transcribed and used for qRT‐PCR using SYBR® Green Pro Taq HS (AG11701, Accurate Biotechnology (Hunan) Co., Ltd., Changsha, China). Semi‐quantitative PCR was performed as previously described (Zhang et al., 2011). Specific primer pairs were designed using Primer Premier 5 (Lalitha, 2004) to produce 80–200 bp amplicons (Table S11). *GhUBQ7* was used as the internal control for normalization in cotton (Nie et al., 2021), and *NtL25* was used as the reference gene in tobacco. The semi‐quantitative RT‐PCR reaction products were separated by gel electrophoresis in a 1% agarose gel (Bio‐Rad Laboratories, Hercules, CA, USA) in 1 × TAE (Tris, acetic acid, ethylendiaminetetraacetic acid).

### Interactions between lncRNAs and miRNAs

MicroRNA target genes were predicted using the psRNATarget web server (http://plantgrn.noble.org/psRNATarget/). lncRNAs were predicted as eTMs of miRNAs using psRobot software (http://omicslab.genetics.ac.cn/psRobot) and identified as described by Wu et al. (2013). The miRNA precursors were predicted as described by Ke et al. (2019).

A microscale thermophoresis assay was performed to verify the interactions between lncRNA67 and miR3367 (Wang et al., 2022b). Sixteen samples with constant concentrations of lncRNA67 (ligand) and two‐fold increased concentrations of CY5‐labeled miR3367 probe (target) were measured using a Monolith NT.115 instrument (NanoTemper, Munich, Germany). Temperature‐induced changes in fluorescence were detected as a function of the concentration of the miR3367 target probe in glass capillaries. Finally, the dissociation constant (*K*_*d*_) was calculated and fitted using NanoTemper Analysis software. Three independent measurements were analyzed. The promoter region of modA was used as the negative‐control ligand.

### Analysis of pollen fertility

Iodine‐KI staining was used to assess pollen fertility in cotton, while pollen germination experiments were used to assess pollen fertility in tobacco. I_2_‐KI solution was used at a concentration of 1% (Niu et al., 2023). The pollen germination medium was described by Song et al. (2014).

### Vector construction and transformation of *N. tabacum* and *N. benthamiana*

A total of 379 bp of conserved fragments of the *GhCYP724B* and *NtCYP724B1*‐like coding sequences were cloned into the pKANNIBAL intron vector. An intermediate vector with a strong CaMV *35S* promoter was ligated to the pART27 binary vector. The recombinant plasmid was transformed into *N. tabacum* plants using the leaf disk method.

The *cox4* gene encodes a mitochondrial leading peptide. The *Aorf27* gene encodes a mitochondrion‐specific protein in the sterile line 2074A. To generate the overexpression constructs, the full‐length coding sequence of *cox4*‐*Aorf27* was inserted into the pCAMBIA2301 vector. Tobacco plants overexpressing the *cox4*‐*Aorf27* gene were generated using Agrobacterium‐mediated transformation of tobacco (*N. tabacum* cv. Xanthi‐nc) leaf disks.

Recombinant plasmids containing the *lncRNA67*, *mlncRNA67*, *miR3367*, and *GhCYP724B* sequences were infiltrated into *N. benthamiana* leaves during the seedling stage. The leaves were sampled 2 d after infiltration to detect GFP signals and to extract RNA.

### Virus‐induced gene silencing

A cotton leaf crumple virus‐based vector, effective in silencing endogenous genes in cotton, was used for virus‐induced gene silencing (VIGS) (Gu et al., 2014). Agrobacterium strains containing pCLCrVB and pCLCrVA recombinant vectors were mixed with an equal volume of infection medium (10 mmol/L MgCl_2_, 10 mmol/L 2‐[*N*‐morpholino]ethanesulfonic acid, and 200 μmol/L acetosyringone). After a 3‐h incubation at 28°C, the mixed suspensions were infiltrated into mature cotton cotyledons using a needleless syringe. The inoculated cotton seedlings were grown in a greenhouse at 25–30°C in the daytime and 20–25°C at night (16/8 h light/dark cycle).

### Clustered regularly interspaced short palindromic repeats/Cas9 expression vector construction and transformation

Using the web tool CRISPR‐P (http://cbi.hzau.edu.cn/crispr/), two sgRNA sequences were designed to target the exons of *lncRNA67* and *GhCYP724B*: sgRNA1/sgRNA2 and sgRNA3/sgRNA4, respectively. sgRNA1 and sgRNA2 were designed to target the first exon of *lncRNA67*, and the binding sites of miR3367 overlapped with the sgRNA1 sequence. Both sgRNA3 and sgRNA4 were located at the conserved functional sites and targeted the two copies of *GhCYP724B* in the A*t* and D*t* subgenomes. The recombinant plasmids were inserted into the multiple cloning sites of the CRISPR/Cas9 vector pRGEB32‐GhU6.9 (Wang et al., 2017c). The hypocotyls of upland cotton cultivar Jin668 were used as explants for tissue culture and transformation (Jin et al., 2006a, 2006b; Tian et al., 2015). In total, six *GhCYP724B* mutants (GM07, GM08, GM19, GM18, GM24, and GM29) and three *lncRNA67* mutants (LM04, LM12, and LM13) were subjected to phenotypic analysis. GM07, GM19, LM04, and Jin668 were used as samples for RNA‐seq.

### Analysis of BR metabolites

Brassinosteroids pathway intermediates were detected using UPLC–MS/MS. For each sample, 0.50 g of flower buds were collected at different stages, from the pollen mother cell stage to the late UNP stage, and BRs were extracted from the samples with acetonitrile (ACN). After filtering through a 0.22 μm membrane filter, the mixed sample was analyzed by UPLC–MS/MS as previously described (Yu et al., 2018).

### Transcriptome analysis

Total RNA was extracted from the flower buds (at the pollen mother cell to late UNP stage) and growing point of leaves in transgenic and WT cotton. The mRNA libraries were prepared and sequenced by Novogene Bioinformatics Institute (Beijing, China) on an Illumina NovaSeq sequencer. The RNA‐seq reads were aligned to the TM‐1 reference genome (Hu et al., 2019) using TopHat2 (Trapnell et al., 2012). The differential expression of each gene was calculated by quantifying the Illumina reads based on FPKM values. Genes with a fold change ≥1.5 or ≤0.67 between samples and a *P*‐value < 0.05 were considered to be DEGs.

### Subcellular localization and co‐localization assays

Recombinant plasmids containing *GFP* or *35S*:*GHCYP724B‐GFP* were transformed into *N. benthamiana* leaves via particle bombardment. The ORFs of *GhDIM* and *GhCYP90B* without the termination codons were amplified and cloned into the pGD‐mCherry vectors. The GFP and red fluorescent protein (RFP) recombinant vectors were infiltrated into *N. benthamiana* leaves at a 1:1 ratio. After 24 h of culture in the dark and 48 h in the light, the fluorescent signals were detected under a fluorescence microscope (FV10‐ASW; Olympus).

### Bimolecular fluorescence complementation assay

The genes of interest were individually cloned into n‐YFP and c‐YFP vectors and co‐expressed in *N. benthamiana* leaves. After 2–3 d of incubation, the YFP fluorescent signals were detected under a fluorescence microscope (FV10‐ASW; Olympus).

### Luciferase complementation imaging assay

An LCI assay was used to explore the interaction between GhCYP724B and Aorf27. Briefly, the full‐length coding sequences of *GhCYP724B* and *Aorf27* were cloned into the JW771 and JW772 vectors, respectively. The recombinant vectors were transformed into Agrobacterium strain GV3101 cells, which were resuspended in infiltration buffer (optical density at 600 nm = 0.6), mixed at a 1:1 ratio (v/v), and infiltrated into the leaves of *N. benthamiana* plants. The LCI assay was conducted as previously described (Ye et al., 2021).

### Yeast two‐hybrid assay

The Y2H assay was performed using the Matchmaker Gold Y2H System (Takara Bio, Kusatsu, Japan) according to the manufacturer's protocol. The full‐length *GhCYP724B* and *Aorf27* sequences were cloned into the pGBDT7 and pGADT7 vectors at the *Eco*RI and *Bam*HI sites, respectively. Various combinations of bait and prey plasmids were co‐transformed into yeast strain Y2H Gold. The yeast cultures were grown on synthetic defined (SD)/−Leu/−Trp medium for 3 d. To test the interaction between the two proteins, the colonies were picked and grown on two plates (SD/−Trp/−Leu/His and SD/−Trp/−Leu/−His/−Ade). Photographs were taken after 5–7 d of incubation at 30°C.

### Expression of *Aorf27* in yeast cells

The full‐length sequence of *Aorf27* was amplified from 2074A cDNA by PCR. The PCR products were cloned into the yeast overexpression vector p426HXT7, and the recombinant plasmid was transformed into yeast (*Saccharomyces cerevisiae*) cells. The empty vector p426HXT7 was used as the control. The yeast cells were grown on SD medium (uracil‐deficient yeast nitrogen‐base; Becton Dickinson, Franklin Lakes, NJ, USA) containing 5 mmol/L (NH_4_)_2_SO_4_ as an N source and cultured in an incubator at 30°C. The primers used to construct the expression vectors are shown in Table S12.

### Rapid amplification of cDNA 5′ ends

A 5′‐RACE assay was performed as previously described (Cao et al., 2020). Briefly, an RNA oligo adaptor was ligated to total RNAs extracted from 2074A flower buds for reverse transcription (Thermo Fisher Scientific, Waltham, MA, USA). For the first round of PCR, the 5′‐RACE outer primer together with the *GhCYP724B‐*specific outer primer were used. Nested PCR amplification was performed to identify the cleavage site in *GhCYP724B* cDNA using the 5′‐RACE inner primer and *GhCYP724B*‐specific inner primer (Table S12). Finally, the amplified product was detected using agarose gel electrophoresis.

### Statistical analysis

Each graph represents the results from at least three repeats; the values are shown as the means ± *SD*. Statistical significance was determined using Student's *t*‐test. * and ** indicate significant differences at *P* = 0.05 and *P* = 0.01, respectively.

## CONFLICTS OF INTEREST

The authors declare no conflict of interest.

## AUTHOR CONTRIBUTIONS

A.G., H.N., C.C., and H.L. performed the experiments. H.N., A.G., H.L. and C.C. analyzed the data. A.G., C.C., B.L., K.J., N.Z., and S.Z. attended discussion and bench work. A.G. and H.N. prepared the manuscript. J.H. conceived and designed the experiments. J.H. revised the manuscript. All authors approved the final manuscript.

## Supporting information

Additional Supporting Information may be found online in the supporting information tab for this article: http://onlinelibrary.wiley.com/doi/10.1111/jipb.13802/suppinfo

**Figure S1.** Comparative analysis of anther development between cytoplasmic male sterile (CMS) line 2074A and its maintainer 2074B in upland cotton
**Figure S2.** Physiological indexes of flower buds in 2074A and 2074B
**Figure S3.** Systematic identification and characteristics of long non‐coding RNAs (lncRNAs) in upland cotton
**Figure S4.** Screening for the candidate long non‐coding RNAs (lncRNAs) related to male sterility
**Figure S5.** Verification of tissue‐specific expression long non‐coding RNAs (lncRNAs) during different stages in different tissues of cytoplasmic male sterile (CMS) and maintainer lines
**Figure S6.** A full view of the interaction network between bud‐specific long non‐coding RNAs (lncRNAs) and brassinosteroids (BRs) metabolism‐related genes
**Figure S7.** *lncRNA67* acts as eTM (endogenous target mimic) of miR3367
**Figure S8.** Silenced *GhChlI* lead to photobleaching phenotype in upland cotton
**Figure S9.** Expression pattern analysis of long non‐coding RNAs (lncRNAs) and their corresponding targets in cytoplasmic male sterile (CMS) line 2074A and its maintainer 2074B in cotton
**Figure S10.** Functional and expression analysis of *lncRNA67* and its target genes in cotton
**Figure S11.** The gene editing types and phenotype of *GhCYP724B* and *lncRNA67* mutants in cotton
**Figure S12.** Off‐target prediction and identification in *GhCYP724B* clustered regularly interspaced short palindromic repeats/CRISPR‐associated protein 9 (Cas9) transgenic plants
**Figure S13.** *GhCYP724B* and *lncRNA67* mutated plants showed dwarf phenotype in cotton
**Figure S14.** Knockout *Gossypium hirsutum* cytochrome P724B (GhCYP724B) causes male sterility in cotton
**Figure S15.** Homology analysis of *Gossypium hirsutum* cytochrome P724B (GhCYP724B) protein with other known CYP724B1 proteins
**Figure S16.** Phylogenetic analysis of *Gossypium hirsutum* cytochrome P724B1 (GhCYP724B1) protein in different species
**Figure S17.** Knocking‐down *CYP724B* in tobacco leads to brassinosteroids (BRs) deficiency symptoms and male semi‐sterility
**Figure S18.** The candidate proteins interact with *Gossypium hirsutum* cytochrome P724B (GhCYP724B), which were predicted using String software
**Figure S19.** Transcriptome sequencing analysis of flower bud in *GhCYP724B* mutants, *lncRNA67* mutant and wild type cotton
**Figure S20.** Promoter regions of genes in regulating pollen development contain DNA elements of E‐box and BR response elements (BRREs)
**Figure S21.** The interaction networks between mitochondrial functional genes (MT genes) and 848 differentially expressed genes (DEGs) in flower bud
**Figure S22.** Phenotype of flowers and filaments in overexpressed *Aorf27* tobacco
**Figure S23.** Overexpressed *Aorf27* decreased pollen viability and pollen germination rate in tobacco

**Table S1.** Long non‐coding RNAs (lncRNAs) identified in upland cotton
**Table S2.** Summary of public Illumina RNA sequencing (RNA‐seq) data used in the present study
**Table S3.** Tissue specifically and differentially expressed long non‐coding RNAs (lncRNAs) in 2074A and 2074B
**Table S4.** Expression correlation of 54 long non‐coding RNAs (lncRNAs) and 54 brassinosteroid (BR)‐related genes in upland cotton
**Table S5**. Long non‐coding RNAs (lncRNAs) act as precursors of microRNAs (miRNAs) in upland cotton
**Table S6.** *miRNAs* target to *lncRNAs* in upland cotton
**Table S7.** Long non‐coding RNAs (lncRNA) acts as eTMs (endogenous target mimics) of microRNA (miRNA)
**Table S8.** Brassinosteroid (BR) metabolite contents in *GhCYP724B* mutants and *lncRNA67* mutants of upland cotton
**Table S9.** 848 co‐existant differentially expressed genes in GM07 vs. wild type (WT), GM19 vs. WT, and LM04 vs. WT comparative compositions in cotton flower bud
**Table S10.** Overexpressed *Aorf27* in tobacco reduced the number and weight of seeds per pod
**Table S11.** Primers for quantitative reverse transcription – polymerase chain reaction (qRT‐PCR) in the present study
**Table S12.** Primers for vector construction and detection

## Data Availability

All raw sequences and processed data for lncRNA sequencing of flower buds in 2074A and 2074B have been deposited in the National Center for Biotechnology Information Sequence Read Archive (SRA) under BioProject ID: PRJNA1167799. Raw data for transcriptome sequencing of flower buds in *GhCYP7248* mutants, *lncRNA67* mutants and WT (Jin668) have been deposited in SRA under BioProject ID: PRJNA1166841.
