# Supplementary material for: The miR3367–lncRNA67–GhCYP724B module regulates male sterility by modulating brassinosteroid biosynthesis and interacting with Aorf27 in Gossypium hirsutum
Source: J Integr Plant Biol. 2024 Nov 11;67(1):169–90. doi: 10.1111/jipb.13802 (PMC11734110; doi:10.1111/jipb.13802)
Supplement: Supplementary file 1 — Figure S1. Comparative analysis of anther development between cytoplasmic male sterile (CMS) line 2074A and its maintainer 2074B in upland cotton Figure S2. Physiological indexes of flower buds in 2074A and 2074B Figure S3. Systematic identification and characteristics of long non‐coding RNAs (lncRNAs) in upland cotton Figure S4. Screening for the candidate long non‐coding RNAs (lncRNAs) related to male sterility Figure S5. Verification of tissue‐specific expression long non‐coding RNAs (lncRNAs) during different stages in different tissues of cytoplasmic male sterile (CMS) and maintainer lines Figure S6. A full view of the interaction network between bud‐specific long non‐coding RNAs (lncRNAs) and brassinosteroids (BRs) metabolism‐related genes Figure S7. lncRNA67 acts as eTM (endogenous target mimic) of miR3367 Figure S8. Silenced GhChlI lead to photobleaching phenotype in upland cotton Figure S9. Expression pattern analysis of long non‐coding RNAs (lncRNAs) and their corresponding targets in cytoplasmic male sterile (CMS) line 2074A and its maintainer 2074B in cotton Figure S10. Functional and expression analysis of lncRNA67 and its target genes in cotton Figure S11. The gene editing types and phenotype of GhCYP724B and lncRNA67 mutants in cotton Figure S12. Off‐target prediction and identification in GhCYP724B clustered regularly interspaced short palindromic repeats/CRISPR‐associated protein 9 (Cas9) transgenic plants Figure S13. GhCYP724B and lncRNA67 mutated plants showed dwarf phenotype in cotton Figure S14. Knockout Gossypium hirsutum cytochrome P724B (GhCYP724B) causes male sterility in cotton Figure S15. Homology analysis of Gossypium hirsutum cytochrome P724B (GhCYP724B) protein with other known CYP724B1 proteins Figure S16. Phylogenetic analysis of Gossypium hirsutum cytochrome P724B1 (GhCYP724B1) protein in different species Figure S17. Knocking‐down CYP724B in tobacco leads to brassinosteroids (BRs) deficiency symptoms and male semi‐sterility Figure S [file JIPB-67-169-s001.doc]

## Supplementary Figures

**
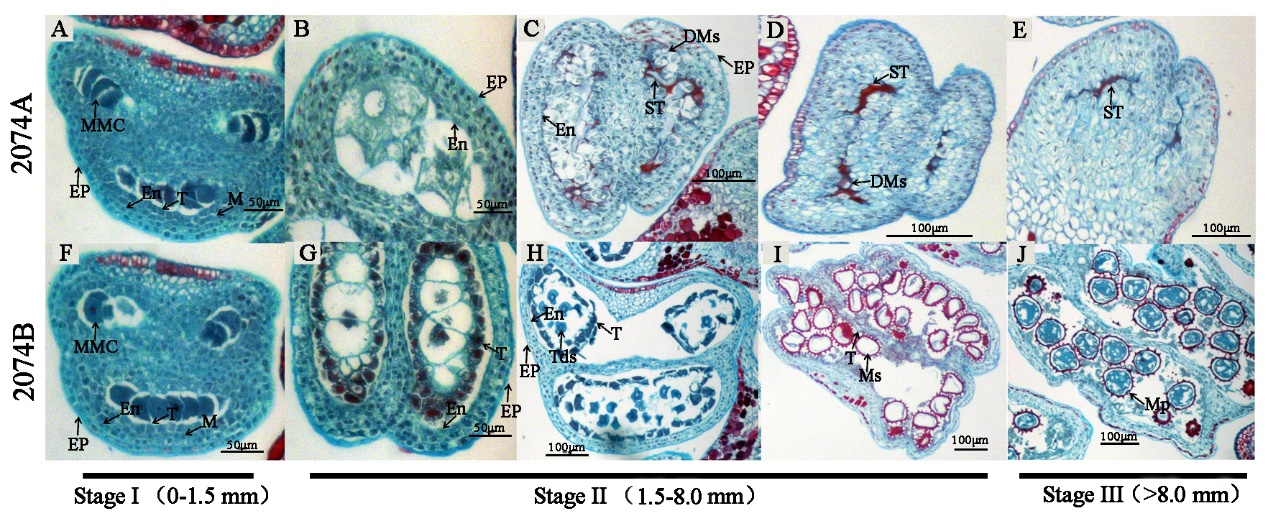
****Figure S1. Comparative analysis of anther development between CMS line 2074A and its maintainer 2074B in cotton.**

Ep: epidermis; En: endothecium; M: middle layer; T: tapetum; MMC: microspore mother cell; Tds: tetrads; Ms: microspore; DMs: degenerated microspores; ST: swollen tapetum; Mp: mature pollen.

**
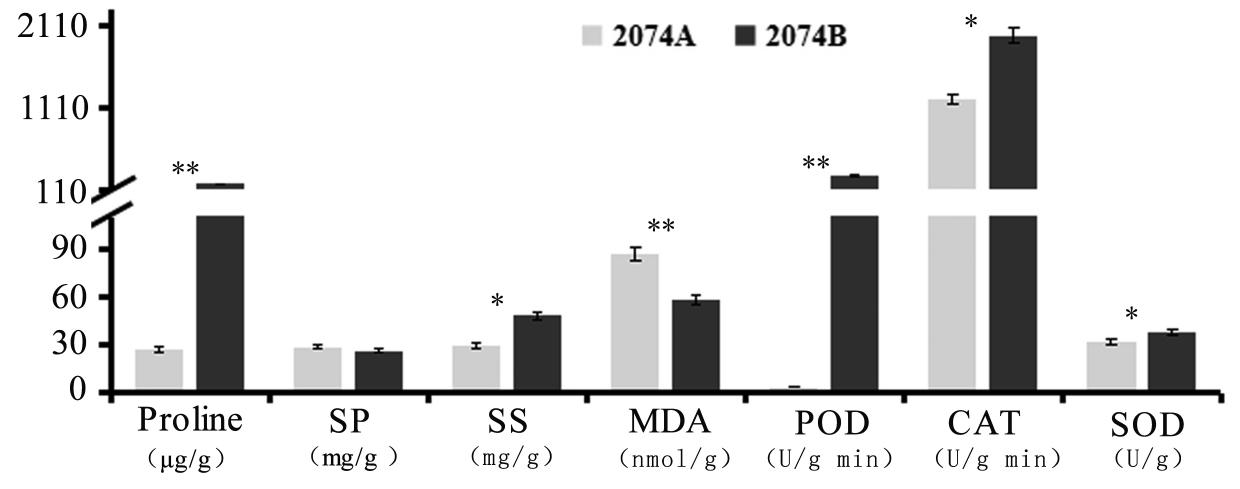
**

**Figure S2. Physiological indexes of flower buds in 2074A and 2074B.**

*, ** represent significant difference at p < 0.05 and at p < 0.01, respectively. The unit for the y-axis was placed under the corresponding physiological indexes.


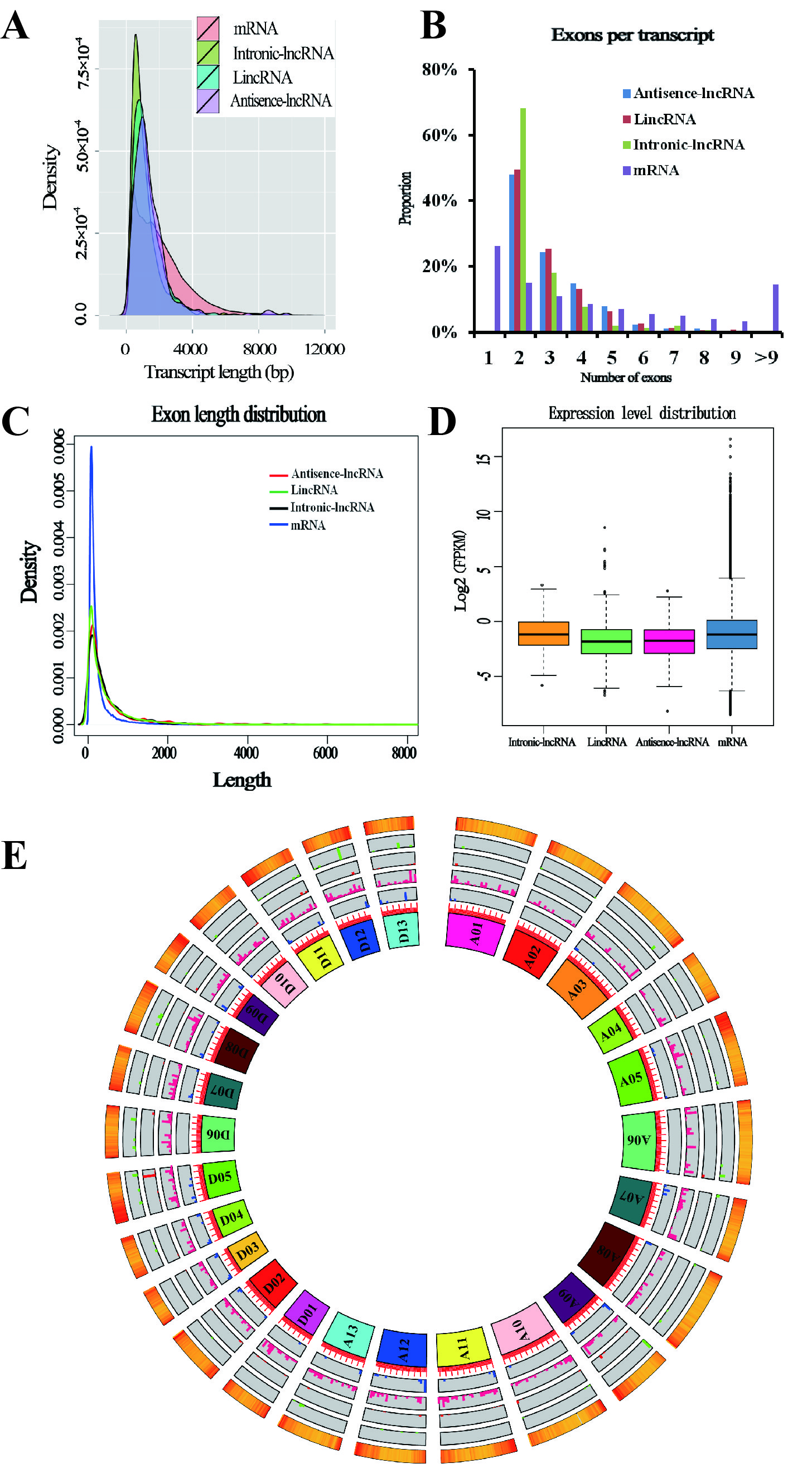


**Figure S3. Systematic identification and characteristics of lncRNAs in upland cotton.**

(A) Length distribution of lncRNA and mRNA transcripts. (B) Number of exons in lncRNA and mRNA transcripts. (C) Distribution of length of exons in lncRNA and mRNA transcripts. (D) Comparative analysis the expression abundance of lncRNAs and mRNAs. (E) Chromosome-wise distribution of miRNAs (II), lincRNA (III); intronic-lncRNA (IV); and antisense-lncRNA (V).


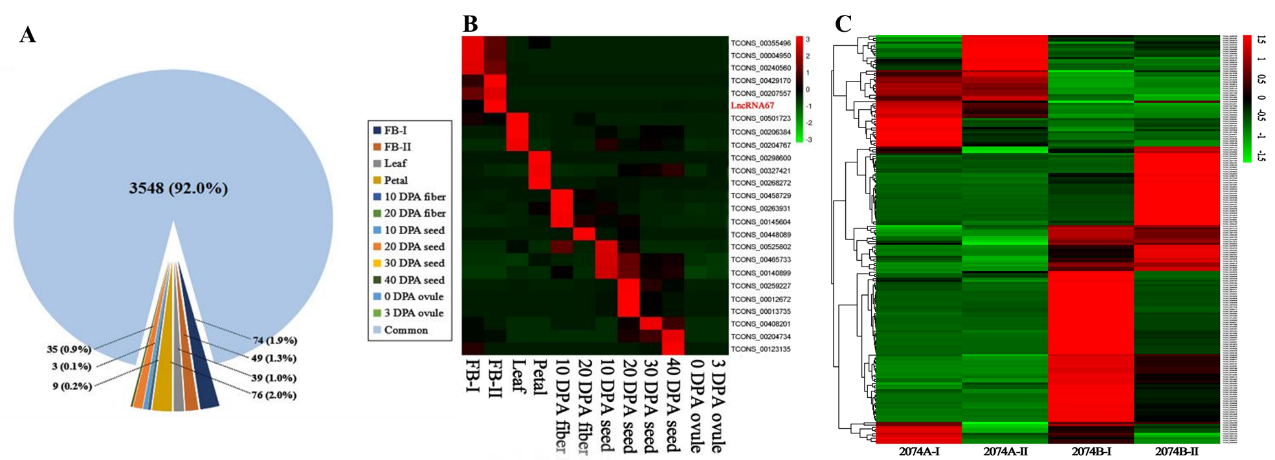


**Figure S4. Screening for the candidate lncRNAs related to male sterility.**

(A) Proportion of lncRNAs exhibiting tissue-specific expression in different tissues. (B) Heat map of lncRNAs which with tissue specific expression. (C) The heat map of different expression lncRNAs between 2074A and 2074B.


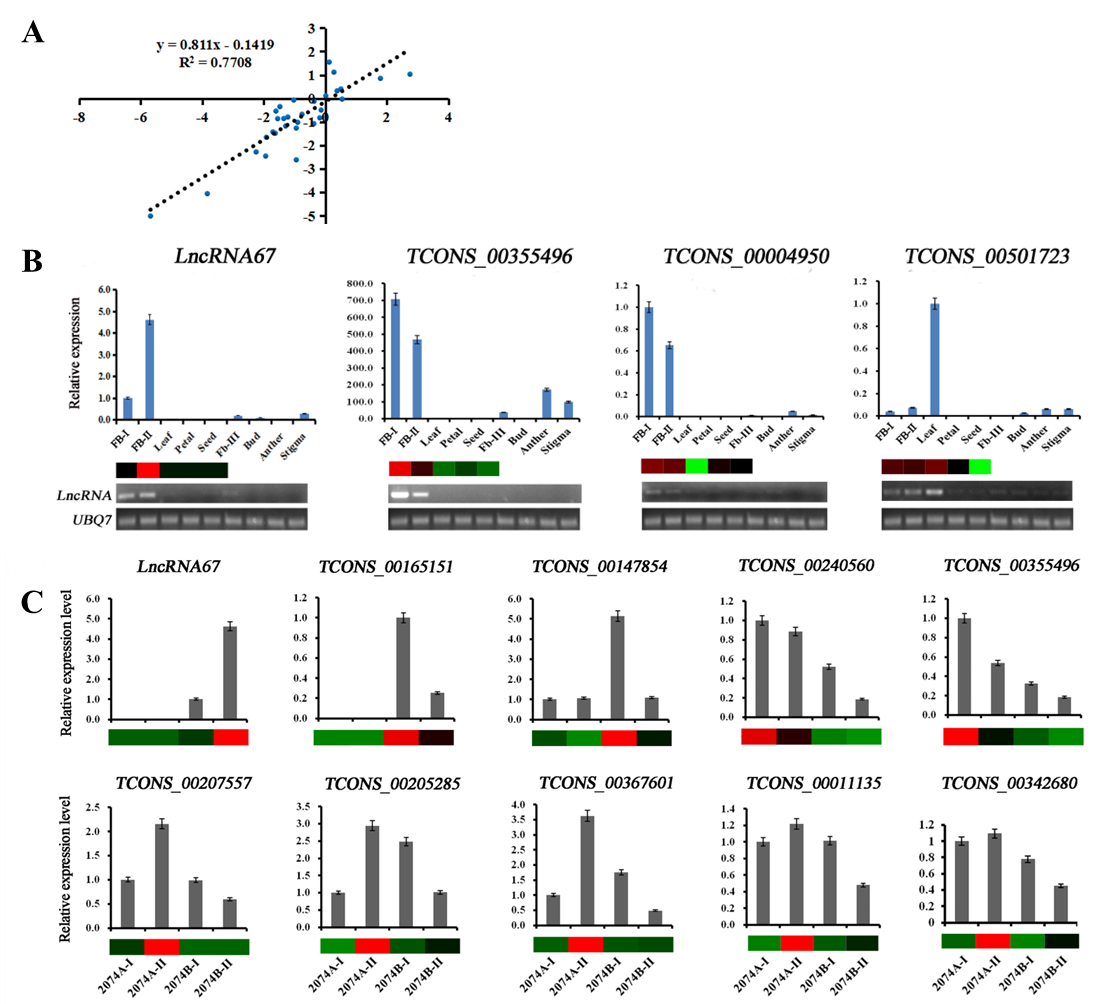


**Figure S5. Verification of tissue-specific expression lncRNAs during different stages in different tissues of CMS and maintainer lines.**

(A) Correlation of the lncRNAs expression profile between the results of sequencing and RT-qPCR. (B) Verification of tissue-specific expression lncRNAs by qRT-PCR and semi-qPCR. FB-I: flower buds at sporogonium stage of 2074B; FB-II: flower buds at pollen mother cells to late UNP stage of 2074B; FB-III: flower buds at the binucleate pollen stage of 2074B; other tissues also sampled form 2074B. (C) The expression pattern of candidate lncRNAs during different stages in CMS and maintainer lines flower bud, the bars represent the results of RT-qPCR, the heat maps indicate the results from sequencing.


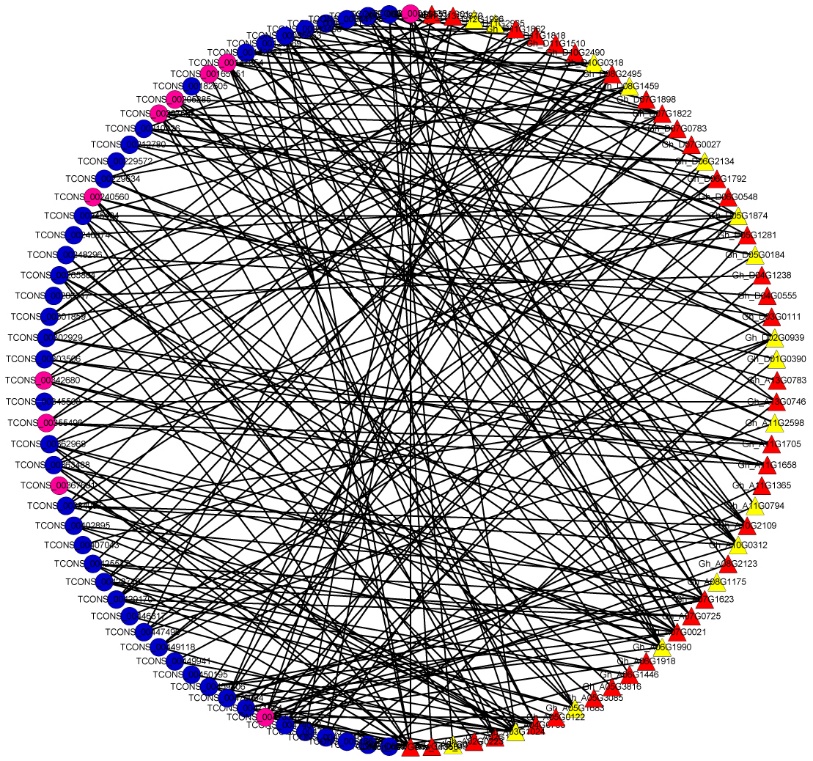


**Figure S6. A full view of the interaction network between bud specific lncRNAs and BRs metabolism-related genes.**


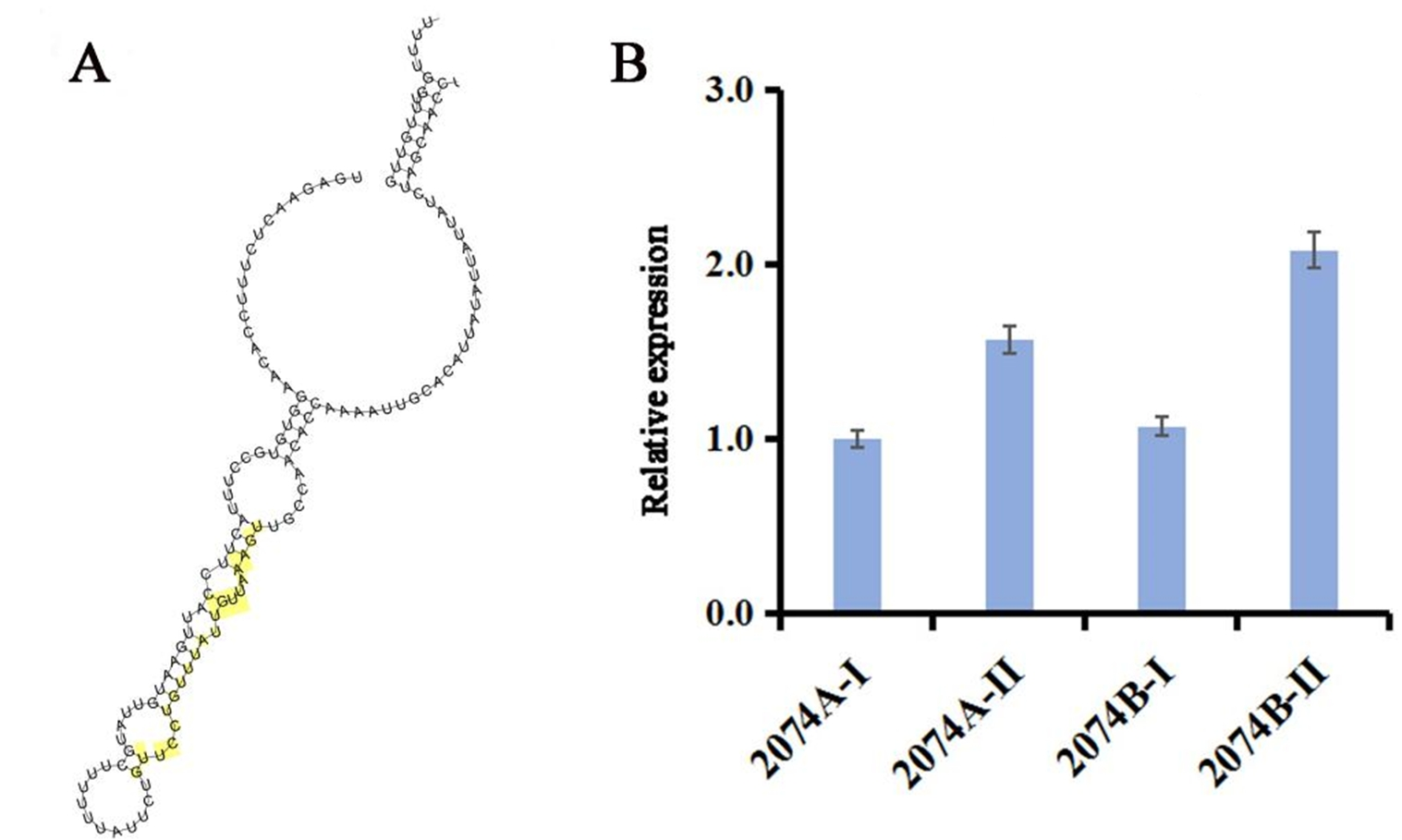


**Figure S7. *LncRNA67* acts as eTM of miR3367.** (A) Stem loop structure of the precursor sequence of miR3367. (B) Expression pattern analysis of miR3367 in 2074A and 2074B flower buds. 2074A-I, 2074A-II, 2074B-I, and 2074B-II, see legend in Figure 1.


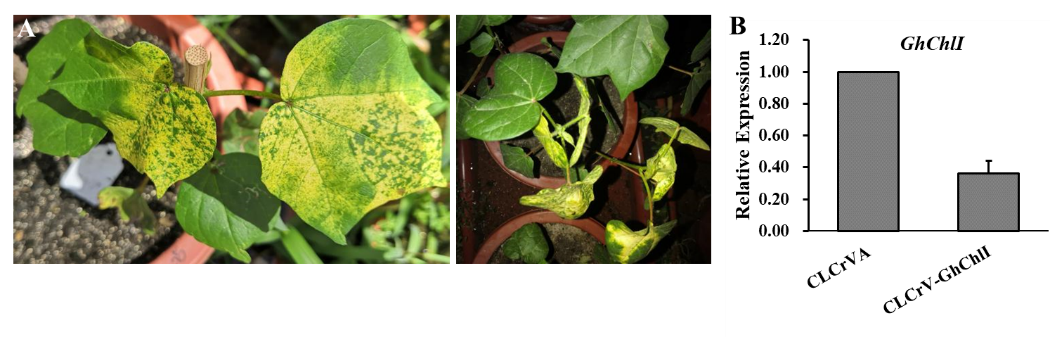


**Figure S8 Silenced *GhChlI* lead to photobleaching phenotype in upland cotton**

(A) Photobleaching phenotypes of *GhChlI*-silenced plants (B) Gene silencing efficiency of *GhChlI* in *GhChlI*-silenced plants.


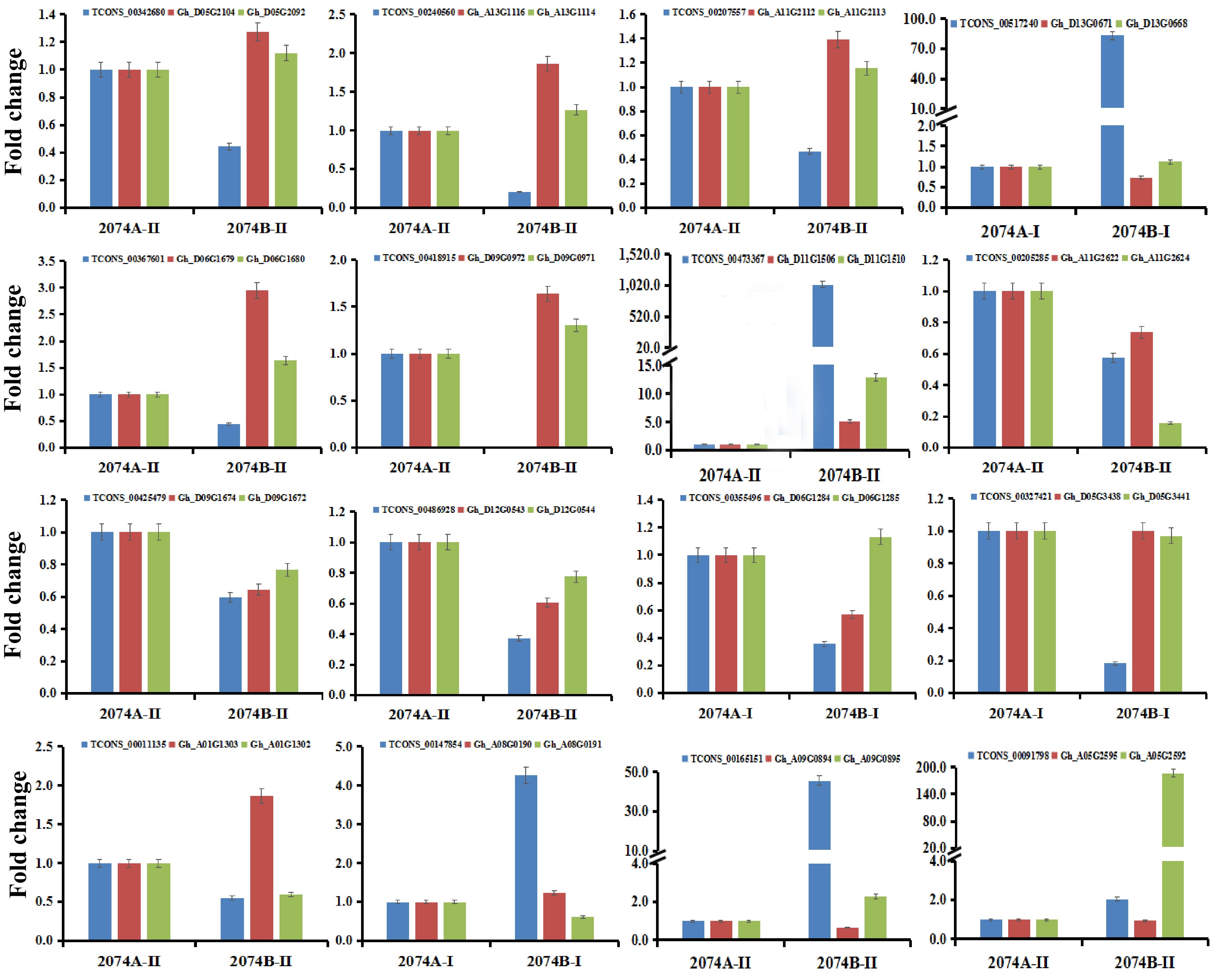


**Figure S9. Expression pattern analysis of lncRNAs and their corresponding targets** **in CMS line 2074A and its maintainer 2074B in cotton.**

2074A-I, 2074A-II, 2074B-I, and 2074B-II, see legend in Figure 1.


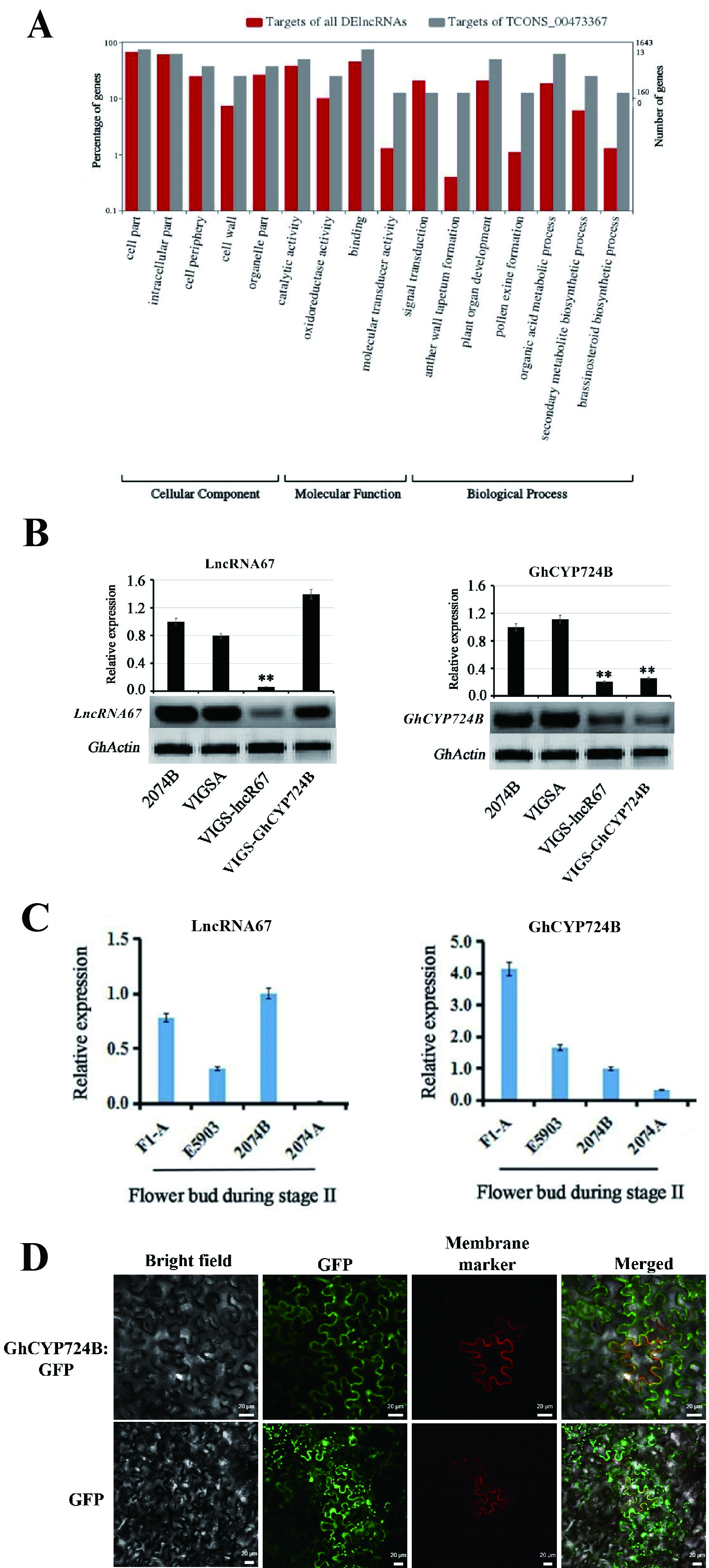


**Figure S10. Functional and expression analysis of *lncRNA67* and its target genes in cotton.**

(A) GO terms of *lncRNA67* target protein coding genes. (B) Expression pattern of *lncRNA67* and *GhCYP724B* in VIGS plants. (C) Expression analysis of *lncRNA67* and *GhCYP724B* in different materials. E5903: the restorer line; F1-A: hybrid (2074A×E5903). (D) Subcellular localization of *GhCYP724B.*


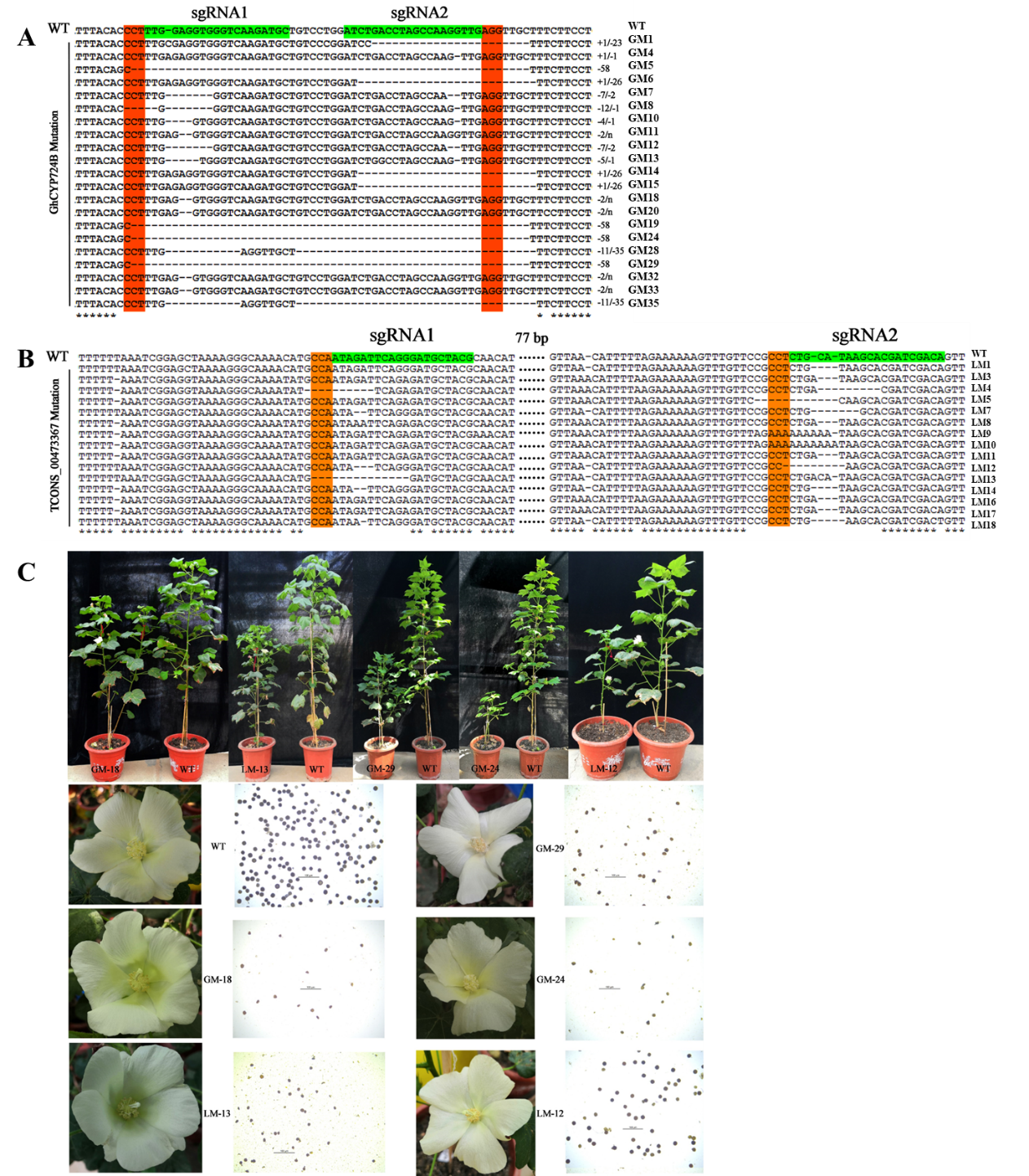


**Figure S11. The gene editing types and phenotype of *GhCYP724B* and *lncRNA67* mutants in cotton.**

(A) The genome editing types at sgRNAs sites of *GhCYP724B*. (B) The genome editing types at sgRNAs sites of *lnR67*.The sgRNA target sites and the PAM regions are highlighted in the green and orange (red) background, respectively. Nucleotide deletions or insertions are labeled at right. (C) The flower and pollen phenotypes of GhCYP724B and lncR67 mutants in cotton. Pollen activity detection using I_2_-KI staining method.


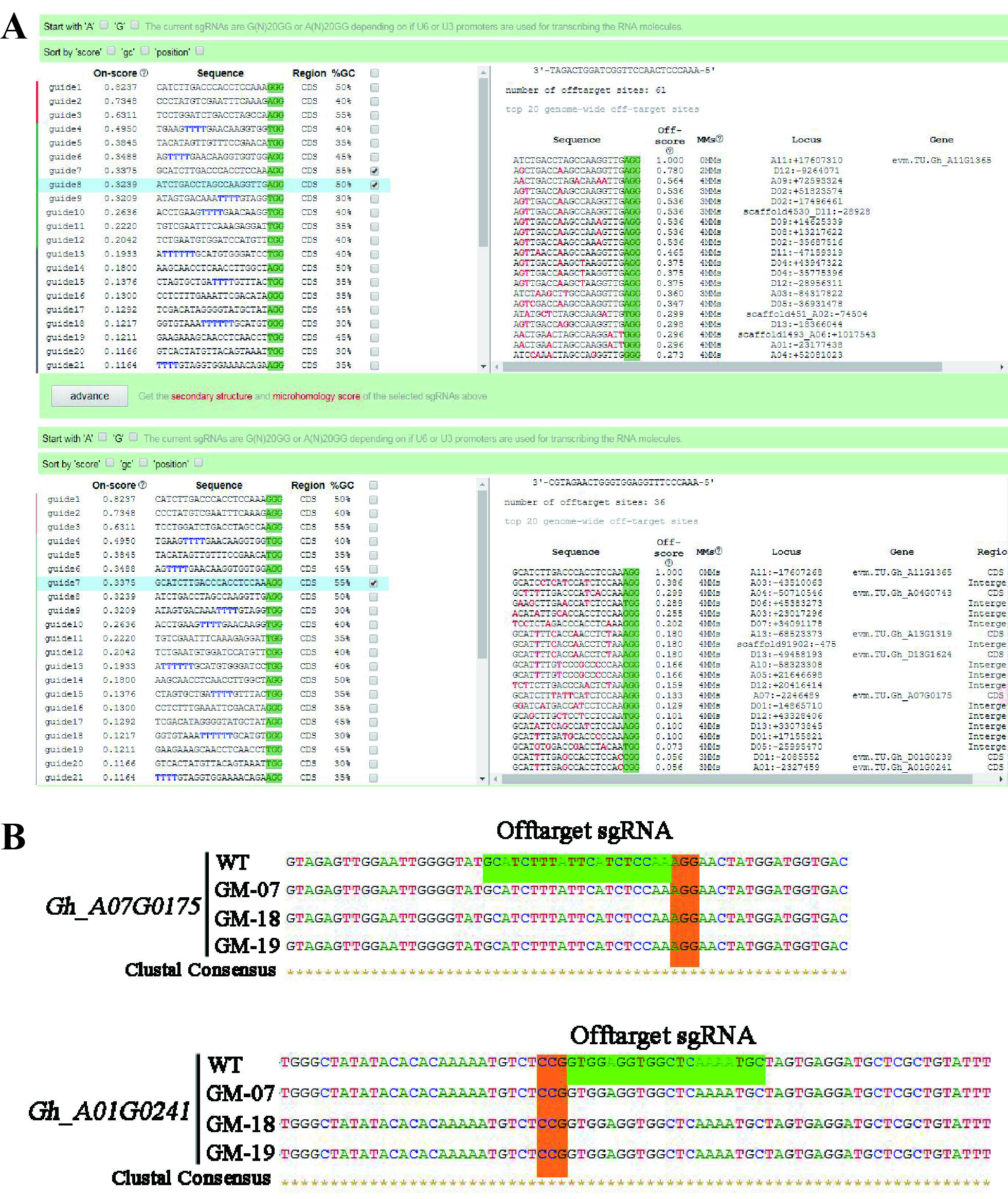


**Figure S12. Off-target prediction and identification in *GhCYP724B* CRISPR/Cas9 transgenic plants.**

Off-target sites were predicted using the web tool CRISPR-P (http://cbi.hzau.edu.cn/crispr/).

**
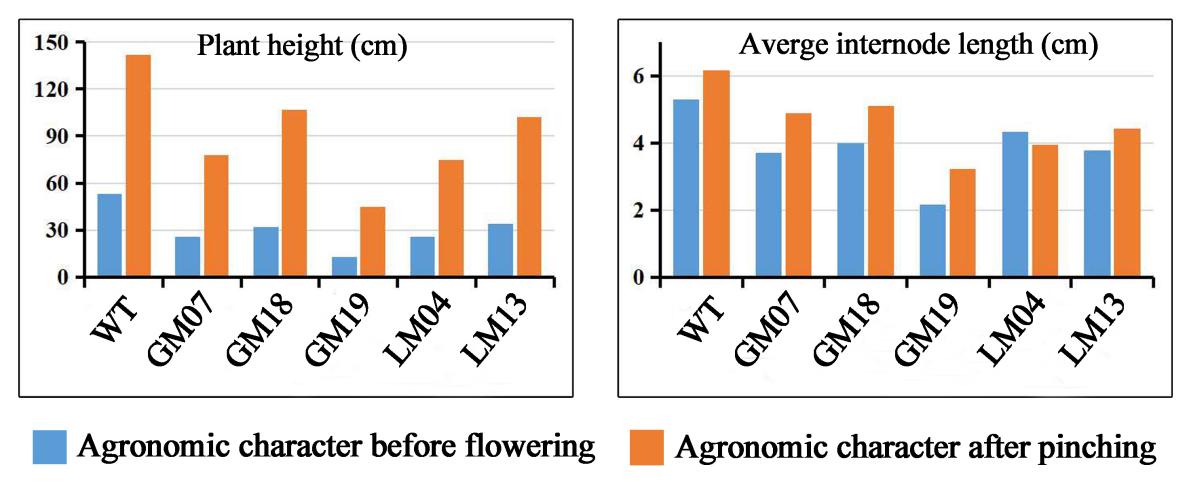
**

**Figure S13. *GhCYP724B* and *lncRNA67* mutated plants showed dwarf phenotype in cotton.**


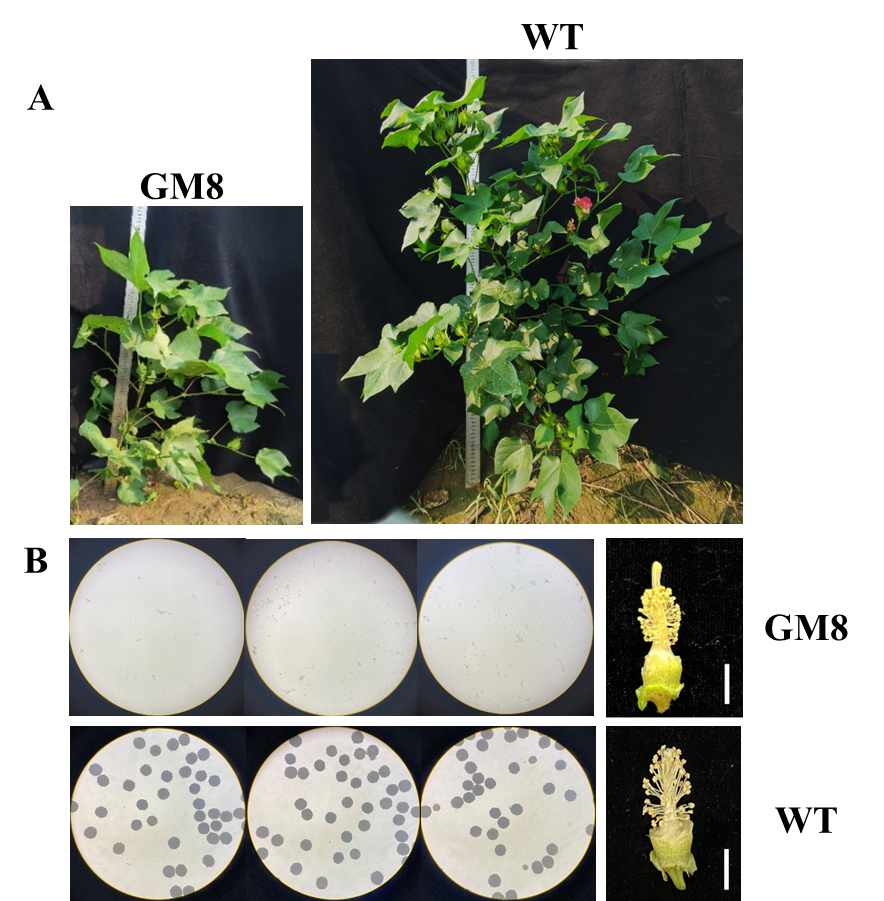


**Figure S14. Knockout GhCYP724B causes male sterility** **in cotton.**

(A) The phenotype of GhCYP724B mutant GM8 (T_4_ generation) and the WT (Jin668) in the field in Hejian, Hebei province; (B) The phenotype of the anthers and I_2_-KI staining of pollen in GhCYP724B mutant GM8 (T_4_ generation) and WT (Jin668); Bar=1 cm.

**
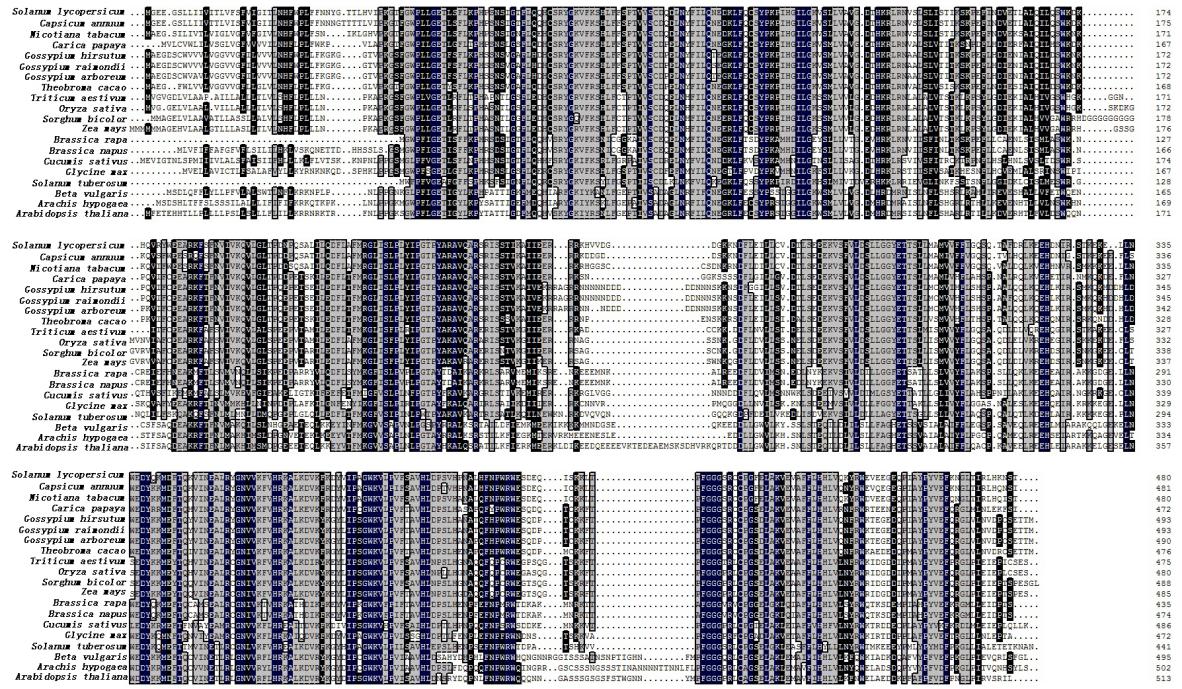
**

**Figure S15. Homology analysis of GhCYP724B protein with other known CYP724B1 proteins.**

**
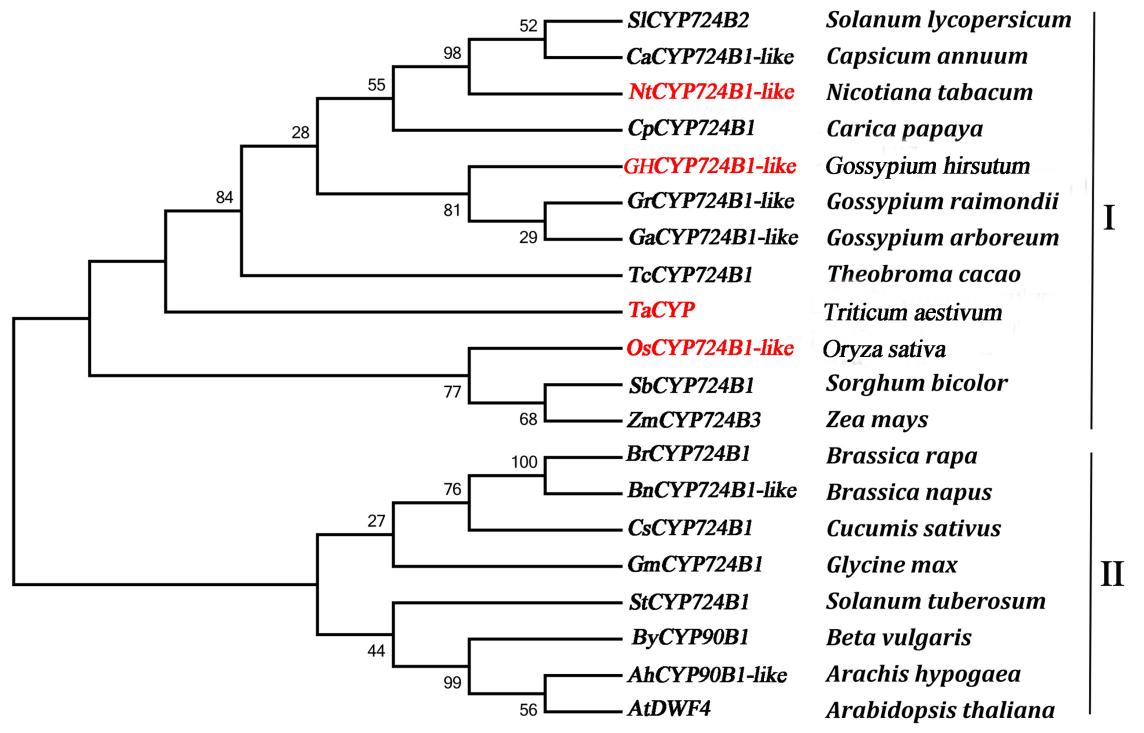
**

**Figure S16. Phylogenetic analysis of CYP724B1 protein in different species.**

Phylogenetic tree was generated using MEGA5.0 software with the neighbor-joining (NJ) method, the statistical significance of the NJ tree typology was evaluated by bootstrap analysis with 1000 replicates. Total of two groups were divided of CYP724B1 proteins between 20 different species, group I include the CYP724B1 from *G. hirsuum*, *O. satia*, *T. aestium*, *N. tabacum* and so on, group II the CYP724B1 from *A. thaliana*, *G. max*, and*B. rapa,* etc.


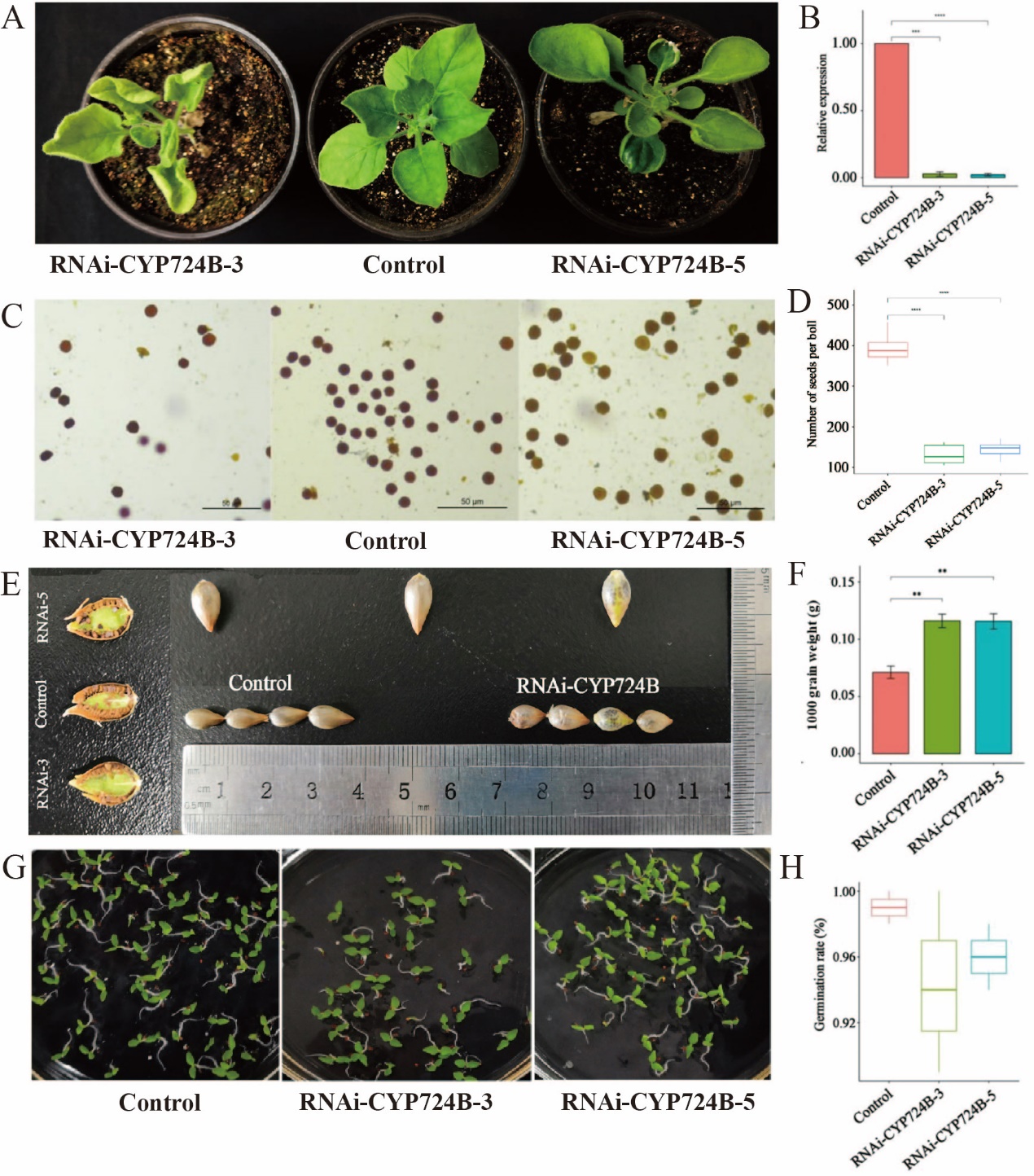


**Figure S17. Knock-downing *CYP724B* in tobacco leads to BRs deficiency symptoms and male semi-sterility.**

(A) Leaves were curly in RNAi-CYP724B tobacco plants. (B) Expression analysis of *CYP724B* in the flower bud of control and RNAi-CYP724B tobacco plants, *NtL25* gene was used as an internal control. (B) Comparison of transverse section and length of the bud in RNAi-CYP724B plants and control plants. (C) Microscopic analysis of pollen grains stained with I_2_-KI solution in the transgenic and control plants (Scale bars: 50 μm). (D) The number of seed per boll of the control and RNAi-CYP724B tobacco plants. (E) The seed number of seed per boll of the control and RNAi-CYP724B tobacco plants. (F) The 1000 grain weight in the control and RNAi-CYP724B tobacco plants. (G) Seed germination in transgenic plants (RNAi-CYP724B-3 and RNAi-CYP724B-5) and control plants on 1/2 MS medium. (I) Germination rate of transgenic plants (RNAi-CYP724B-3 and RNAi-CYP724B-5) and control plants on 1/2 MS medium. **, *** show a significant difference at p < 0.01 and p < 0.001, respectively.

**
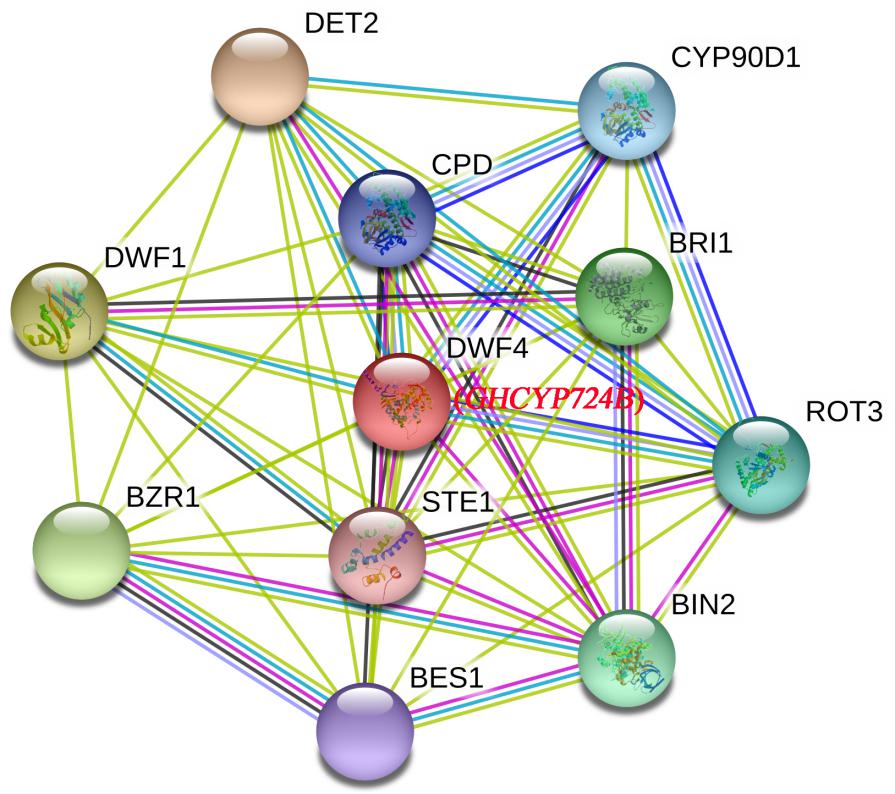
**

**Figure S18. The candidate proteins interact with GhCYP724B predicted using String software.**


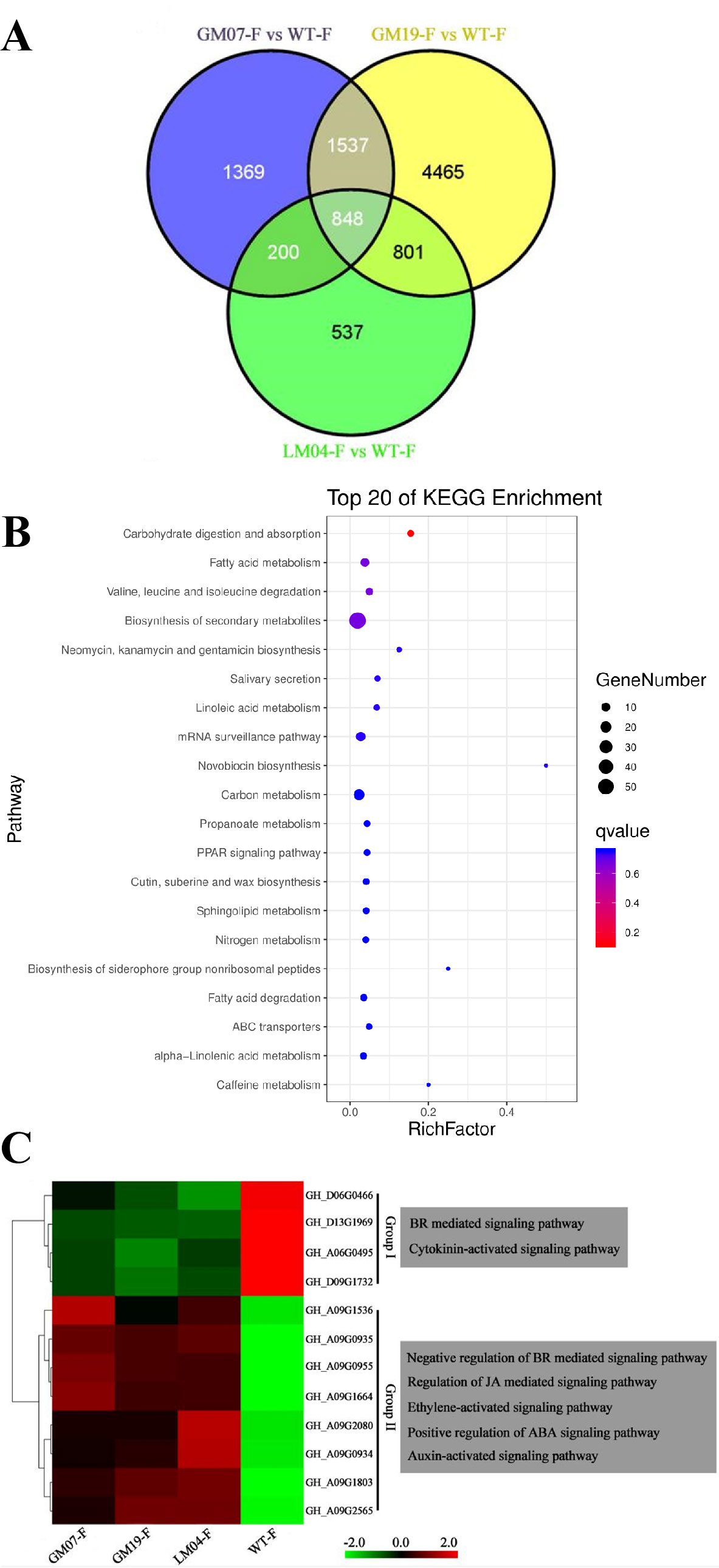


**Figure S19. Transcriptome sequencing analysis of flower bud in *GhCYP724B* mutants, *lncRNA67* mutant and wild type cotton.**

(A) Co-existed DEGs in GM07-F vs WT-F, GM19-F vs WT-F and LM04-F vs WT-F comparative compositions. GM07-F, GM19-F, LM04-F, and WT-F: flower bud of GM07, GM19, LM04, and WT. (B) KEGG enrichment analysis of 848 co-existed DEGs. (C) The expression pattern of genes which related to hormone biosynthesis and signal transduction.


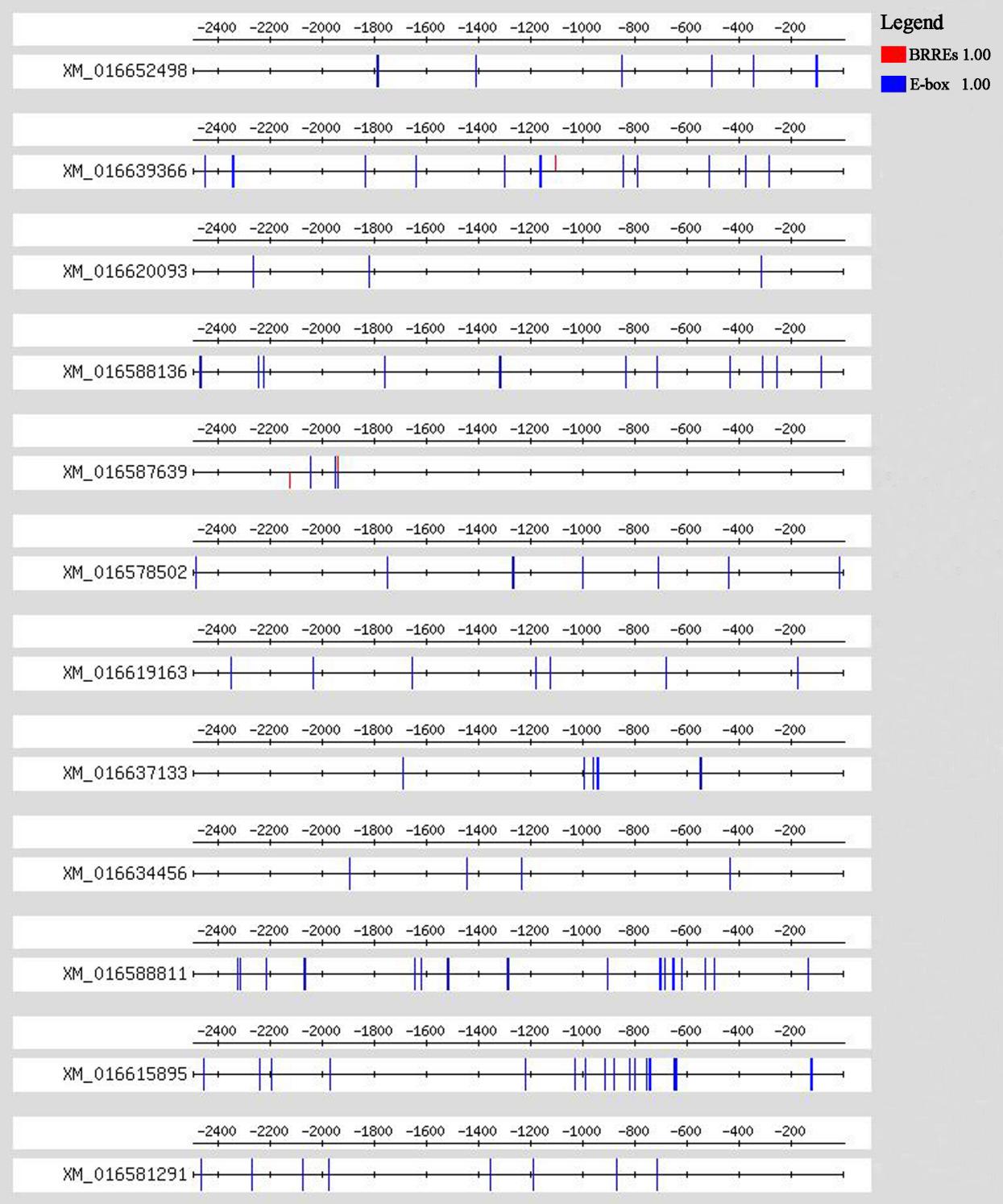


**Figure S20. Promoter regions of genes in regulating pollen development contain DNA elements of E-box and BRREs.**

Blue bars indicate E-boxes, and red bars indicate BRREs. Two-thousand and five hundred base pair upstream sequences from the start codon of each gene were analyzed using the “Regulatory Sequence Analysis Tools” (http://rsat.ulb.ac.be/rsat/).


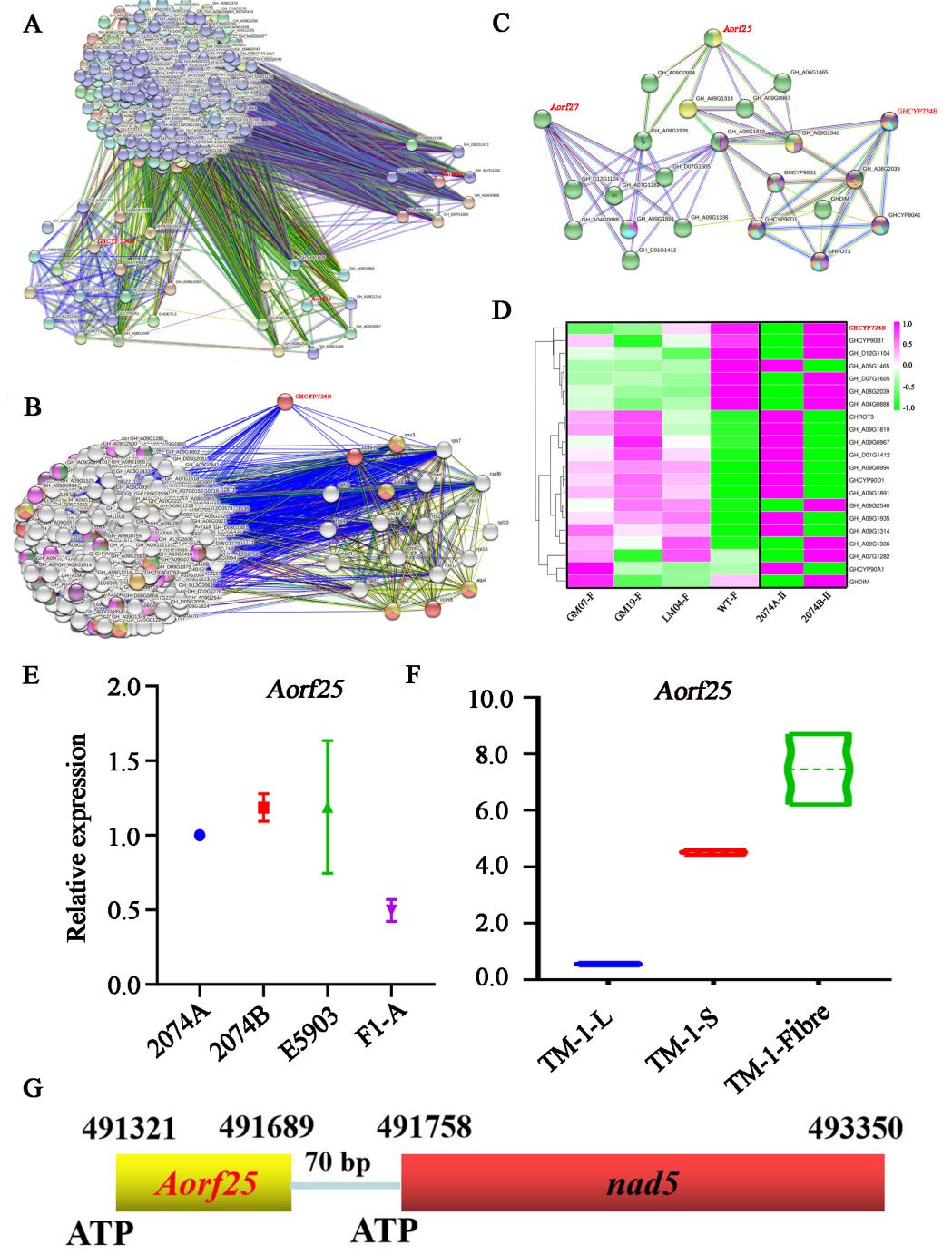


**Figure S21. The interaction networks between MT genes and 848 DEGs in flower bud.**

(A) Two novel ORFs in 2074A interact with 848 DEGs. (B) Mitochondrial functional genes interact with 848 DEGs. (C) GHCYP724B interacts with Aorf25 and Aorf27 indirectly. (D) Expression pattern of the proteins which interacted with mitochondrial ORFs. (E) qRT-PCR analysis of *Aorf25* expression in 2074A, 2074B, E5903 and F_1_A floral buds (1.5-8.0 mm). E5903, the restorer line; F_1_-A, three-line hybrid. (F) Expression pattern analysis of *Aorf25* in the leaf, seed and fibre of TM-1. (G) Genomic structures of *Aorf25* in cotton species.

*
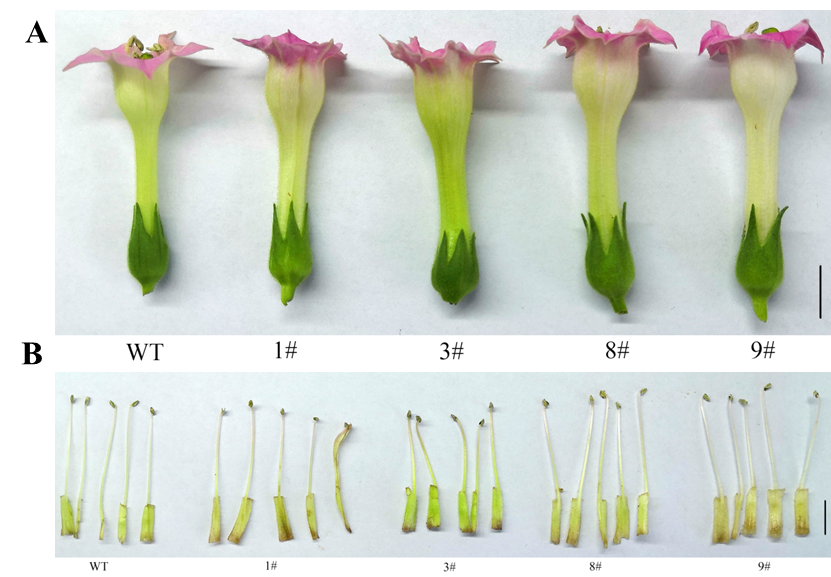
*

**Figure S22. Phenotype of flowers and filaments in overexpressed *Aorf27* tobacco.**

Flowers (A) and filaments (B) in overexpressed *Aorf27* tobacco. Bar=1 cm.


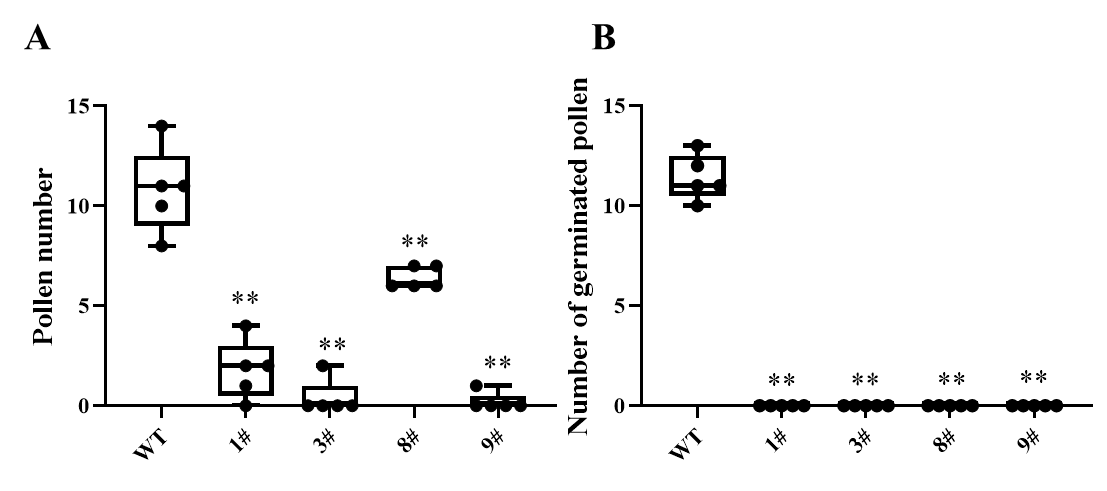


**Figure S23. Overexpressed *Aorf27* decreased pollen viability and pollen germination rate in tobacco.**

(A) Statistical analysis of fertile pollen in *Aorf27* overexpressed tobacco. Two anthers per flower were used in the I_2_-KI staining. Each field of view is an image magnified by 400 under a light microscope. (B) Statistical analysis of germinated pollen. Each field of view is an image magnified by 400 under the light microscope.
